# Surgical, perventricular, and transcatheter closure for ventricular septal defects: a network meta-analysis of randomized controlled trials

**DOI:** 10.1186/s12872-026-05794-w

**Published:** 2026-03-28

**Authors:** Oscar Rivera-Torrejon, Lelis G. Coronel-Chucos, Diego Chambergo-Michilot, Mario Enrique Diaz-Barrera, Rubí Paredes-Angeles, Alvaro Taype-Rondan

**Affiliations:** 1 Universidad de Piura, Lima, Peru; 2 Facultad de Ciencias de la Salud, Universidad Continental, Lima, Peru; 3 Universidad Científica del Sur, Lima, Peru; 4 Nova Evidence Research Group, Trujillo, Peru; 5 Unidad de Investigación para la Generación y Síntesis de Evidencias en Salud, Universidad San Ignacio de Loyola, Lima, Peru

**Keywords:** Ventricular septal defect, Transcatheter closure, Perventricular closure, Surgical repair, Network meta-analysis

## Abstract

**Background:**

Ventricular septal defect is a common congenital heart defect. While surgical repair has been the standard, transcatheter and perventricular closures have emerged as minimally invasive alternatives.

**Methods:**

We conducted a systematic review and network meta-analysis of randomized controlled trials. We searched PubMed, Embase, Cochrane Library, clinicaltrials.gov, and Google Scholar up to February 2026. Risk of bias was assessed using the Cochrane tool, and the certainty of evidence was evaluated with GRADE. We conducted pairwise random-effects meta-analyses for direct comparisons, followed by a frequentist random-effects network meta-analysis to indirectly compare transcatheter and perventricular closure.

**Results:**

Seven randomized controlled trials involving 2,126 participants were included. Studies were conducted in China, Egypt, or Russia. No deaths were reported. Transcatheter and perventricular closures may reduce blood transfusion needs compared to surgical repair, but the evidence is very uncertain. Perventricular closure may reduce hospital stay and operative time but may increase tricuspid regurgitation risk, compared to surgical repair. No significant differences were observed in indirect comparisons of transcatheter and perventricular approaches. Certainty of the evidence was low to very low.

**Conclusion:**

Current evidence suggests that transcatheter and perventricular approaches may be associated with a lower need for blood transfusion compared with surgical repair, while perventricular closure may also reduce length of hospital stay but may increase tricuspid regurgitation. However, the evidence is uncertain, precluding firm conclusions and highlighting the need for well-designed, adequately powered randomized controlled trials.

**PROSPERO I Registration:**

CRD42024595282

**Supplementary Information:**

The online version contains supplementary material available at 10.1186/s12872-026-05794-w.

## Introduction

Ventricular septal defect (VSD) is one of the most common congenital heart defects, characterized by an abnormal opening in the interventricular septum that allows communication between the ventricles. Depending on its size and location, a VSD may require closure to prevent complications such as heart failure, pulmonary hypertension, and endocarditis [1, 2]. The three primary approaches for VSD repair are conventional surgical repair (CSR), transcatheter device closure (TDC), and perventricular device closure (PDC). While CSR has traditionally been the gold standard, TDC and PDC techniques have gained prominence due to their minimally invasive nature [3, 4].

CSR involves direct repair of the septal defect using a synthetic patch or autologous tissue. Although highly effective, this approach requires open-heart surgery with cardiopulmonary bypass, which carries risks such as infection, prolonged recovery, and postoperative complications.

TDC is a catheter-based technique that allows defect repair without the need for open surgery. Guided by echocardiographic or fluoroscopic imaging, a closure device is delivered through a catheter to seal the defect [5, 6]. In contrast, PDC combines aspects of both CSR and TDC techniques. It is performed via a small incision in the chest without cardiopulmonary bypass, offering a less invasive alternative while maintaining direct surgical control [7].

Two previous systematic reviews with network meta-analyses (NMAs) have compared these three techniques [8, 9]. However, they did not assess the certainty of the evidence and combined randomized controlled trials (RCTs) with non-randomized studies, factors that may compromise the reliability of their findings and highlight the need for a more comprehensive and rigorous analysis.

This systematic review aims to synthesize evidence from RCTs comparing CSR, TDC and PDC techniques for VSD repair.

## Methods

### Study design and registration

We performed a systematic review, following the PRISMA 2020 extension statement for reporting of systematic reviews incorporating NMAs [10] (Supplementary Material 1). We registered our review on PROSPERO (https://www.crd.york.ac.uk/prospero/) with the identifier number CRD42024595282.

### Research question and eligibility criteria

We included RCTs published in scientific journals, excluding conference abstracts. RCTs were included if they were conducted in children and adolescents (< 18 years) with a diagnosis of congenital VSD, regardless of the type of defect —subarterial, perimembranous, inflow, or muscular— or comorbidities, with indication for surgical closure. Studies that included both adults and children were eligible for inclusion only when more than 50% of participants were under 18 years of age (i.e., when the reported mean age was below 18 years).

Eligible RCTs had to compare any two or all three of the following VSD closure methods: CSR via open-heart surgery, TDC or percutaneous closure using a device (occluder) regardless of size and manufacturer, and PDC or hybrid closure that combines CSR and TDC procedures.

### Search strategy

We systematically searched PubMed, Embase, Cochrane Library, clinicaltrials.gov, and Google Scholar up to February 15, 2026, without restrictions on language or publication date. Additionally, we reviewed clinical trial registries (ClinicalTrials.gov), as well as the reference lists of eligible studies and previous systematic reviews [11–13], to identify any further eligible studies. The complete search strategies for each database are provided in Supplementary Material 2.

### Selection and data collection process

Search results from electronic databases were imported into Rayyan^®^ (Qatar Computing Research Institute, Doha, Qatar), where duplicates were manually identified and removed. Four review authors (DCM, LGCC, MDB, and ORT) worked in pairs to screen studies and extract data. Each pair independently assessed titles and abstracts, followed by full-text screening. Discrepancies were initially resolved within pairs and, if needed, through discussion with the full review team to reach consensus.

For data collection, we developed a data extraction form. Subsequently, four authors (DCM, LGCC, MDB, and ORT) independently extracted data from the included studies in pairs. Any inconsistencies were resolved through discussion, with the involvement of a third review author (ATR) when necessary.

### Variables collected

For each included study, we collected information on study design, funding sources, population characteristics, interventions, comparators, and results. We systematically extracted data on all relevant outcomes, encompassing both continuous and categorical variables. When needed, we contacted the corresponding authors of the papers via email to request additional information.

Adverse events were classified as major or minor according to the definitions used in the primary studies. When more than one time point was available for a given outcome, we used the longest available follow-up.

### Risk of bias assessment

To evaluate the risk of bias, we employed the eight domains of the Cochrane Risk of Bias Tool [14]: random sequence generation, allocation concealment, blinding of participants, blinding of personnel, blinding of outcome assessment, incomplete outcome data, selective reporting, and other potential sources of bias.

In pairs and independently, four authors (DCM, LGCC, MDB, and ORT) evaluated the risk of bias in the included studies. Any disparities were resolved through discussion or by seeking input from a third author (ATR).

### Statistical analyses

We calculated odds ratios (ORs) or mean differences (MDs), along with their 95% confidence intervals (CIs). For dichotomous outcomes, we also estimated risk differences (RDs) by applying the ORs to baseline risks derived from the median risk in the control group. All analyses were conducted using Stata version 17 (StataCorp).

The included studies compared CSR with either TDC or PDC; however, no study directly compared TDC with PDC. Therefore, we initially conducted conventional pairwise meta-analyses of the direct comparisons (CSR vs. TDC and CSR vs. PDC) using a DerSimonian-Laird random-effects model.

Then, a frequentist random-effects NMA was performed using the “network” suite of commands in Stata. We assumed a common between-study heterogeneity variance (τ²) across all treatment comparisons. As no study directly compared TDC and PDC, only indirect comparisons were possible, and statistical assessment of inconsistency (incoherence) could not be performed [15]. For outcomes with zero events in one or both study arms, we applied a continuity correction by adding 0.5 to each cell of the 2 × 2 contingency table to allow estimation of effect sizes.

### Certainty of the evidence assessment

We assessed the certainty of the evidence for all recorded outcomes using the Grading of Recommendations Assessment, Development, and Evaluation (GRADE) approach for NMAs [16, 17], and categorizing it as high, moderate, low, or very low.

To assess the certainty for direct comparisons, we examined potential risk of bias (applying the Cochrane risk of bias tool, evidence was downgraded by one level when most studies had high risk of bias), imprecision (evidence was downgraded by one level when the total number of events was fewer than 50 for dichotomous outcomes or when fewer than 300 participants contributed to continuous outcomes, and by two levels when confidence intervals were very wide and included both clinically important benefit and harm), indirectness (evidence was downgraded when the populations, interventions, comparators, or outcomes of the included studies differed from the review question), inconsistency (evidence was downgraded by one level when substantial heterogeneity was present: I² > 50%), and publication bias (assessed when applicable, generally requiring at least 10 studies for a given meta-analysis; in such cases, funnel plots were planned). For indirect comparisons, we used the lower certainty rating of the direct estimates within the loop and assessed intransitivity [16].

## Results

### Selection of studies

**Fig. 1:**
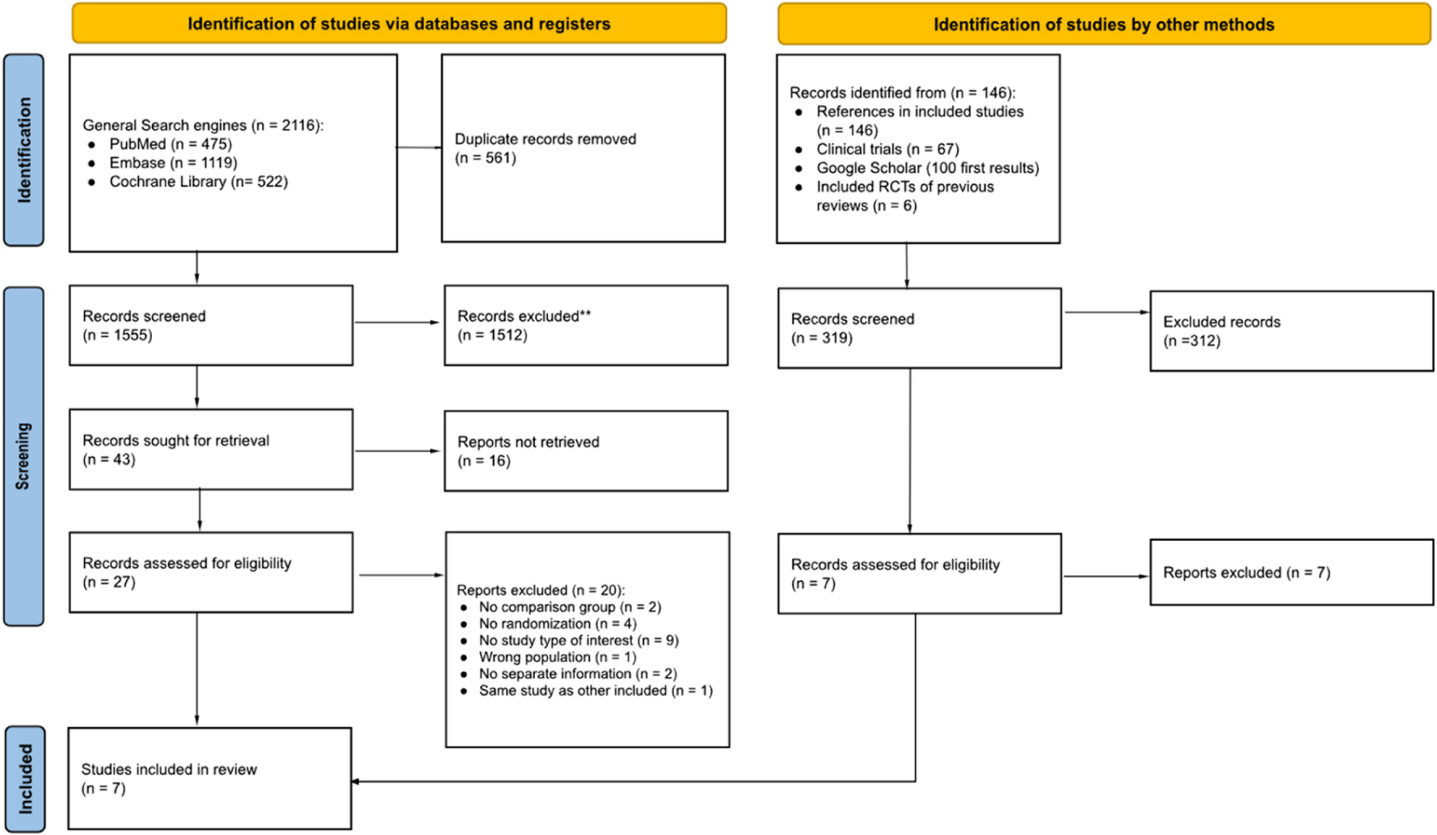
Flowchart of the selection of included studies

The database search yielded 2,116 records. After removal of duplicates, 1,555 records were screened based on titles and abstracts, of which 43 were selected for full-text assessment. Of these, 16 reports were excluded at the full-text screening stage because the full text could not be retrieved; most corresponded to documents published in Chinese journals. Due to the unavailability of full texts, eligibility could not be fully assessed, and these records were therefore excluded from the review. Consequently, 27 full-text documents were assessed for eligibility, and seven studies met the inclusion criteria [18–24]. No additional studies were identified through other sources. The study selection process is illustrated in Fig. 1 and the list of records excluded after full-text review is provided in Supplementary Material 3.

### Study characteristics

The characteristics of the included studies are detailed in 1. Of the seven included RCTs, three compared TDC with CSR, while four compared PDC with CSR of VSD. The studies were conducted between 2014 and 2023 in China, Egypt, and Russia, with populations primarily consisting of pediatric patients. Sample sizes ranged from 41 to 640 participants, and the proportion of female participants varied from 41.9% to 54.2%. The mean age of participants ranged from 14.3 months to 16 years.

**Table 1:** Studies characteristics

Author (Country, year)	Population	Sample size (I/C), age, females	Defect Size (mm)(I/C)	Qp/Qs(I/C)	Comparator(surgical patch)	Intervention (device)	Funding	Maximumfollow-up
Studies comparing TDC vs CSR:								
Shang (China, 2016) [18]	pmVSD and moderate or severe tricuspid regurgitation	N = 41 (21/22)Age: 16 ± 9 yrFemales: 41.9%	I: 6.7 ± 1.9C: 7.4 ± 2.5	NR	● CSR (Dracon patch)● Tricuspid valvuloplasty was done for severe tricuspid regurgitation	TDC (Shanghai pmVSD occluder / Access: NR)	NR	12 mo
Singab (Egypt, 2023) [19]	pmVSD and mVSD	N = 72 (36/36)Age: 5 ± 4 yrFemales: 54.2%	I: 5.4 ± 1.4C: 7.4 ± 2.8	NR	● CSR (1 was repaired with direct closure, 5 with patch closure with autologous pericardium, and 30 with Gortex polyethylene terephthalate patch)● In 1 patient a temporary pacemaker was needed due to complete heart block	● TDC (Amplatzer™, Cera™, KONAR-MF™ / Anterograde and retrograde access)● In 3 patients the procedure failed. One of these patients was referred for elective surgery.	Self-funded by the authors	Until discharge (mean: 4.9 days)
Yang (China, 2014) [20]	pmVSD	N = 229 (114/115)Age: 6 ± 3 yrFemales: 44.6%	I: 5.2 ± 6.1C: 5.9 ± 5.3	I: 2.5 ± 2.7C: 2.3 ± 2.2	● CSR (75.8% were repaired with a Dacron or pericardial patch, while 24.2% underwent primary closure)● In 1 patient a thoracic re-exploration was needed due to post-surgical bleeding	● TDC (Shanghai pmVSD occluder / femoral vein and artery access)● In 3 patients a second occluder was required	Public funding	40 mo
Studies comparing PDC vs CSR:								
Liu (China, 2018) [21]	Isolated pmVSD	N = 200 (100/100)Age: 24 ± 9 moFemales: 52%	I: 5.5 ± 1.4C: 5.5 ± 1.4	I: 2.0 ± 0.2C: 2.0 ± 0.3	CSR (Dracon patch or direct suture)	● PDC (NR)● 3 patients were directly converted to CSR, 1 due to severe aortic regurgitation after deployment of the occluder device, 2 due to complete atrioventricular block.	Multiple public funding sources	Until discharge
Lu (China, 2021) [22]	Isolated pmVSD	N = 100 (50/50)Age: 13 ± 8 yrFemales: 53%	I: 5 ± 1.53C: 6 ± 1.53	NR	● CSR (Dracon patch or direct suture)● Temporary detachment of the tricuspid valve was used.	● PDC (NR)● 1 patient had intraoperative conversion to CSR	Multiple public funding sources	56 mo
Voitov (Russia, 2017) [23]	Isolated VSD (pmVSD, mVSD, sVSD, or reVSD)	N = 640 (320/320)Age: 27 ± 19 moFemales: 46.1%	I: 5.1 ± 1.5C: 6.0 ± 2.1	I: 1.8 ± 0.2C: 1.8 ± 0.3	● CSR (Diepoxide-treated xeno-pericardial patch)● 11 patients initially underwent perventricular closure.	● PDC (MemoPart VSD Occlusion Device)● In patients with re-VSD, a complete resternotomy was used.	NR	mean: 27 mo
Zhang (China, 2015) [24]	pmVSD	N = 530 (265/265)Age: 14.3 ± 8.1 moFemales: 48.3%	I: 7.05 ± 2.42C: 7.24 ± 2.32	NR	● CSR (glutaraldehyde-pretreated autologous pericardium patch).● In two patients, temporary pacing was required due to complete heart block, after which sinus rhythm was restored.	● PDC (Shanghai pmVSD occluder)● 11 patients were converted to CSR	NR	12 mo

*VSD* Ventricular septal defect, *CSR* Conventional surgical repair, *TDC* Transcatheter device closure, *PDC* Perventricular device closure, *pmVSD* Perimembranous ventricular septal defect, *mVSD* Muscular ventricular septal defect, *sVSD* Subarterial ventricular septal defect, *reVSD* Residual ventricular septal defect, *DS* Defect size, *NR* Not reported, *mo* months, *yr* years

### CSR vs. TDC

Three studies [18–20] directly compared TDC with CSR for VSD, involving a total of 344 patients. Defect sizes in the intervention groups ranged from 5.2 mm to 6.7 mm, with similar sizes in the surgical groups. CSR was performed using patch repair (Dacron, autologous pericardium, Gortex) or direct suture, with one study also reporting adjunctive tricuspid valvuloplasty. TDC was achieved using devices such as the Shanghai perimembranous VSD occluder, Amplatzer™, Cera™, and KONAR-MF™, delivered via femoral, anterograde, or retrograde access. Procedural complications were reported in both groups. Follow-up durations ranged from hospital discharge (mean: 4.9 days) to 40 months.

### CSR vs. PDC

Four studies [21–24] compared CSR with PDC, including a total of 1,470 patients. The average VSD size ranged from 5.1 mm to 7.05 mm, with similar measurements across both groups. CSR involved patch closure (Dacron, glutaraldehyde-treated autologous pericardium, xenopericardium) or direct sutures. PDC was performed using various devices (MemoPart, Shanghai occluders), with direct access to the heart through a minimal incision and without cardiopulmonary bypass. Procedural complications were reported in both groups, and follow-up durations varied significantly, ranging from hospital discharge to 56 months.

### Risk of bias

**Fig. 2:**
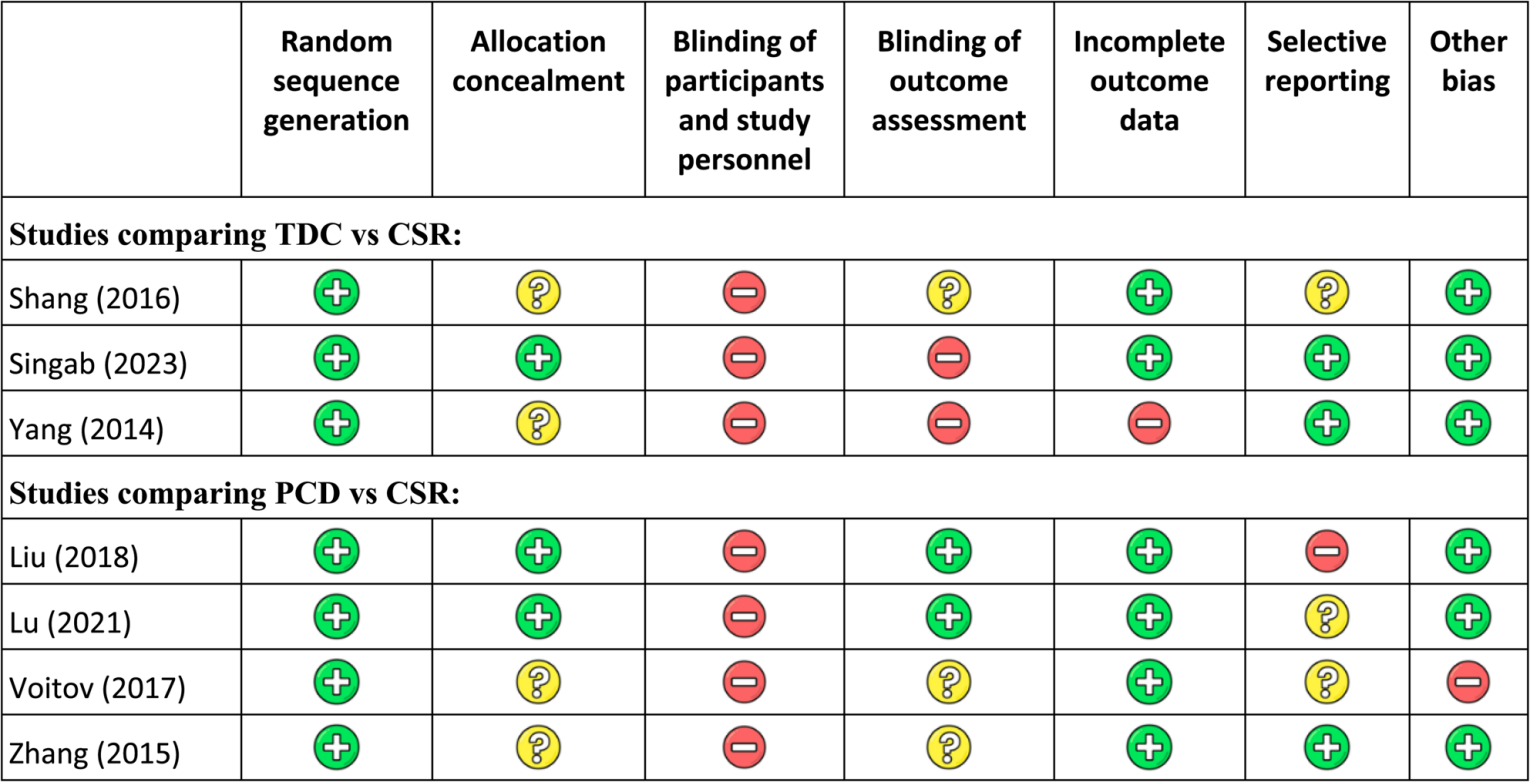
Risk of bias. CSR: Conventional surgical repair, TDC: Transcatheter device closure, PDC: Perventricular device closure. Green circles indicate a low risk of bias, red circles indicate a high risk of bias, and yellow circles indicate an unclear risk of bias

All studies had a low risk of bias for random sequence generation. Allocation concealment was unclear in 4/7 studies. Blinding of participants and personnel was judged at high risk of bias in all studies, while blinding of outcome assessment was at high or unclear risk in 5/7 studies. One study had a high risk of bias due to incomplete outcome data, selective reporting was at high or unclear risk in 4/7 studies, and other sources of bias were judged to be at high risk in one study (Fig. 2).

### Effects of the interventions

**Fig. 3:**
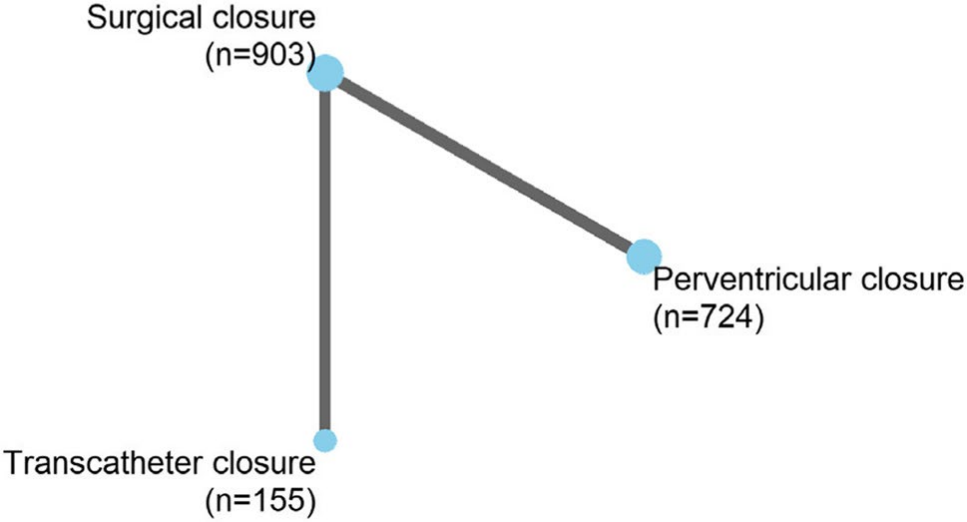
Network plot for complete closure

The summary of findings for the direct and indirect comparisons is presented in Table 2. The corresponding meta-analyses are provided in Supplementary Material 4. The network plot for complete closure (the only outcome reported by all included studies) is shown in Fig. 3, while network plots for the remaining outcomes are available in Supplementary Material 5.

**Table 2:** Summary of findings

Outcomes	I: TDC | C: CSR (only direct estimates) Measures (95% CI)	I: PDC | C: CSR (only direct estimates) Measures (95% CI)	I: PDC | C: TDC (only indirect estimates) Measures (95% CI)
Mortality	Not calculable, as no deaths occurred in the included studies.		
Complete closure	OR: 1.08 (0.35 to 3.34) RD: +4 per 1000 (-94 to + 41) Certainty: Very low (a, b)	OR: 1.13 (0.60 to 2.14) RD: +7 per 1000 (-36 to + 31) Certainty: Very low (a, b)	OR: 1.02 (0.25 to 4.14) RD: -0 per 1000 (-29 to + 8) Certainty: Very low (c)
Major adverse events	OR: 0.73 (0.13 to 4.03) RD: -3 per 1000 (-9 to + 29) Certainty: Very low (a, b)	OR: 1.49 (0.24 to 9.25) RD: +5 per 1000 (-8 to + 75) Certainty: Very low (a, b)	OR: 2.04 (0.17 to 24.85) RD: +7 per 1000 (-6 to + 142) Certainty: Very low (c)
Minor adverse events	OR: 0.34 (0.08 to 1.41) RD: -207 per 1000 (-332 to + 84) Certainty: Very low (a, d, e)	Not calculable, as none of the studies comparing PDC and CSR have assessed this outcome	Not calculable
Tricuspid regurgitation	OR: 1.38 (0.38 to 5.03) RD: +14 per 1000 (-23 to + 128) Certainty: Very low (a, b)	**OR: 2.80 (1.63 to 4.81)** **RD: +62 per 1000 (+ 22 to + 122)** **Certainty: Low (a**,** d)**	OR: 2.03 (0.50 to 8.27) RD: +48 per 1000 (-25 to + 260) Certainty: Very low (c)
Blood transfusion†	**OR: 0.01 (0.00 to 0.07)** **RD: -755 per 1000 (-792 to -582)** **Certainty: Very low (a**,** b**,**f)**	**OR: 0.01 (0.00 to 0.01)** **RD: -755 per 1000 (-792 to -755)** **Certainty: Very Low (a**,** e**, **f)**	OR: 0.16 (0.00 to 11887.16) RD: -1 per 1000 (-999 to 921) Certainty: Very low (c)
Blood loss, in mL	**MD: -164 (-203 to -125)** **Certainty: Low (a**,** d)**	MD: -160 (-440 to 121) Certainty: Low (a, e)	MD: 4 (-483 to 491) Certainty: Very low (c)
Hospital stay, in days	MD: -2.80 (-5.77 to 0.18) Certainty: Very low (a, d, e)	**MD: -4.64 (-8.61 to -0.67)** **Certainty: Low (a**,** e)**	MD: -1.83 (-6.79 to 3.14) Certainty: Very low (c)
Operative time, in minutes	MD: -83 (-199 to 33) Certainty: Low (a, e)	**MD: -94 (-117 to -71)** **Certainty: Low (a**,** e)**	MD: -12.64 (-104.21 to 78.94) Certainty: Very low (c)

Bold: statistically significant (p<0.05)

*I* Intervention group, *C* Control group, *CSR* Conventional surgical repair, *TDC* Transcatheter device closure, *PDC* Perventricular device closure, *OR* Odds Ratio, *RD* Risk difference

†For blood transfusion, in the studies reporting this outcome, no patients in the TDC (0/137) or PDC (0/574) groups required transfusion, whereas 599/731 patients in the CSR group did. These estimates are therefore based on zero-event arms in the minimally invasive groups and should be interpreted with caution due to sparse data. Explanations of the certainty of the evidence: a) Downgraded one level due to risk of bias. b) Downgraded two levels due to Imprecision, as the total number of events was < 50. c) The certainty of the indirect estimates was rated as very low, as no direct comparisons were available to assess incoherence. In addition, concerns regarding risk of bias in the contributing studies and potential differences in age and clinical characteristics across trials further limited confidence in these estimates. d) Downgraded one level due to imprecision, as the total number of events (in dichotomic outcomes) or participants (in numeric outcomes) was < 300. e) Downgraded one level due to heterogeneity, as I2 was > 50%. f) Downgraded by one level for imprecision due to very wide 95% confidence intervals

For the outcome of mortality, no deaths were reported in any of the included studies, preventing any quantitative comparison between groups.

For the outcomes of complete closure and major adverse events, no statistically significant differences were found between TDC and CSR, or between PDC and CSR. Similarly, indirect comparisons between PDC and TDC showed no significant differences. However, the certainty of the evidence was rated as very low for all comparisons, indicating a high level of uncertainty in the findings.

For minor adverse events, no significant differences were observed between TDC and CSR, although the evidence was highly uncertain due to very low certainty. This outcome was not assessed in any of the studies comparing PDC and CSR.

The studies used different criteria to classify major and minor adverse events; these criteria are detailed in Supplementary Material 6.

For tricuspid regurgitation, PDC may increase the risk of presenting tricuspid regurgitation compared to CSR (OR: 2.80; 95% CI: 1.63 to 4.81), with an absolute risk increase of 62 per 1000 (95% CI: from 22 more to 122 more; low certainty of evidence). No significant differences were observed when comparing TDC with CSR, nor when comparing TDC with PDC. Bold: statistically significant (*p* < 0.05). I: Intervention group. C: Control group. CSR: conventional surgical repair. TDC: transcatheter device closure. PDC: perventricular device closure. OR: Odds Ratio. RD: Risk difference. †For blood transfusion, in the studies reporting this outcome, no patients in the TDC (0/137) or PDC (0/574) groups required transfusion, whereas 599/731 patients in the CSR group did. These estimates are therefore based on zero-event arms in the minimally invasive groups and should be interpreted with caution due to sparse data.

For blood transfusion, TDC may reduce the risk of blood transfusion compared to CSR (OR: 0.01; 95% CI: 0.00 to 0.07) with an absolute risk reduction of 755 per 1000 (95% CI: from 792 fewer to 582 fewer; very low certainty of evidence). However, this evidence is highly uncertain. Notably, no patients in the TDC (0/137) or PDC (0/574) groups required transfusion, whereas 599 of 731 patients in the CSR group did. Therefore, these estimates are driven by zero-event arms in the minimally invasive groups and should be interpreted with considerable caution due to sparse data and imprecision.

Similarly, PDC may reduce the risk of blood transfusion compared to CSR (OR: 0.01; 95% CI: 0.00 to 0.01), with an absolute risk reduction of 755 per 1000 (95% CI: from 972 fewer to 755 fewer; very low certainty of evidence), but the evidence is very uncertain. No significant differences were observed when comparing TDC with PDC.

For blood loss, TDC may reduce blood loss compared to CSR (MD: -164 mL; 95% CI: from − 203 mL to -125 mL; low certainty of evidence). No significant differences were observed when comparing PDC with CSR, nor when comparing TDC with PDC.

For hospital stay, PDC may reduce hospital stay compared to CSR (MD: -4.64 days; 95% CI: from − 8.61 days to -0.67 days; low certainty of evidence). No statistically significant differences were observed when comparing TDC with CSR, nor when comparing TDC with PDC.

For operative time, PDC may reduce operative time compared to CSR (MD: -94 min; 95% CI: from − 117 min to -71 min; low certainty of evidence). No significant differences were observed when comparing TDC with CSR, nor when comparing TDC with PDC.

## Discussion

### Summary of main results

The evidence on the effects of TDC, PDC, and CSR remains uncertain across several outcomes. No deaths were reported in any of the included studies, and the effect on complete defect closure, as well as major and minor adverse events, was not statistically significant and was highly uncertain. Compared to CSR, PDC may increase the risk of tricuspid regurgitation, however it may reduce the risk of blood transfusion, shorten hospital stays, and reduce operative time. On the other hand, compared to CSR, TDC may reduce the risk of blood transfusion and may reduce blood loss.

### Study characteristics

Overall, the comparison between CSR and PDC included a greater number of RCTs and more than three times the total number of participants compared with studies evaluating CSR versus TDC. This imbalance may be partly explained by the greater procedural similarity between CSR and PDC, which facilitates trial implementation and may make blinding of surgeons and outcome assessors more feasible. In contrast, the substantial procedural differences between CSR and TDC—particularly regarding access route, use of cardiopulmonary bypass, and imaging guidance—pose greater challenges for trial design, standardization, and execution, thereby limiting the number of randomized comparisons conducted.

The study populations were composed predominantly of young children; however, the trials by Shang [18] and Lu [22] also included adult patients. This may introduce variability in procedural outcomes, as anatomical and physiological characteristics differ across age groups. Despite this heterogeneity, there was relative consistency in VSD size among participants, generally ranging from 5.1 to 7.05 mm, corresponding to small to moderate defects. Notably, no studies included patients with large or severe VSD, likely because such cases usually require surgical repair as the standard of care, which limits their feasibility for inclusion in RCTs.

Age differences across trials represent a plausible source of intransitivity in our network. Some studies predominantly included infants, whereas others enrolled older children and, in two cases, adolescents or adults. Age may influence anatomical characteristics, vascular access feasibility, procedural complexity, and complication profiles (e.g., atrioventricular block or valvular injury), potentially modifying treatment effects [25]. Although we considered exploring age-stratified or meta-regression analyses, these were not feasible due to the small number of trials and limited reporting of age-specific outcome data. Therefore, residual intransitivity related to age cannot be excluded and further reduces confidence in indirect comparisons.

### Certainty of the evidence

The assessment of the certainty of evidence in a NMA is based on the evaluation of both direct and indirect comparisons, as well as the NMA as a whole. However, among the studied comparisons, direct estimates were available only for TDC vs. CSR and PDC vs. CSR, whereas the TDC vs. PDC comparison relied exclusively on indirect estimates derived from the network. The absence of direct randomized comparisons between TDC and PDC means that conclusions regarding their relative effects are based solely on indirect evidence within the network. Therefore, these comparisons should be considered hypothesis-generating rather than practice-changing.

None of the included studies reported blinding of participants or study personnel, and most did not blind outcome assessment. Although this is expected given the inherent challenges of blinding in surgical trials, its absence resulted in a high risk of performance bias. While some outcomes (e.g., transfusion, operative time) are relatively objective, others (e.g., minor adverse events, tricuspid regurgitation grading) could be more subjective and prone to detection bias. Moreover, even “objective” outcomes may be influenced by clinicians’ awareness of the intervention, potentially affecting management decisions. Therefore, the lack of blinding, together with concerns about selective reporting and allocation concealment. led us to downgrade the certainty of the evidence by one level for risk of bias in all direct comparisons.

Among the outcomes assessed, the certainty of evidence for mortality was very uncertain in all comparisons, as no events were recorded across studies. Regarding the comparison of TDC versus CSR, with the exception of the outcomes of blood loss and operating time, the certainty of the evidence for the other outcomes was very low. The reasons for downgrading the certainty of the evidence were mainly due to imprecision and risk of bias.

Regarding the comparison of PDC versus CSR, most outcomes had low certainty of evidence, downgraded mainly due to risk of bias and heterogeneity. The outcomes of complete closure and major adverse events had very low certainty of evidence, mainly due to risk of bias and imprecision. The outcome of minor adverse events was not assessed by the studies.

Regarding the comparison of PDC versus TDC, the certainty of evidence for all outcomes was very low, except for minor adverse events, as it was not calculable. The main reasons for the very low certainty of evidence rating were the initial grading of the certainty of the evidence from direct comparisons of the first-degree loop and the intransitivity due to age differences between the studies.

Although some outcomes were presented as statistically significant in some of the comparisons (blood loss and blood transfusion in the comparison of TDC versus CSR; tricuspid regurgitation, blood loss, blood transfusion, hospital stay, and operative time in the comparison of PDC versus CSR), the presentation of very low to low certainty of evidence should be interpreted as indicating that the evidence is very uncertain for those results.

### Agreements and disagreements with other studies or reviews

Previous systematic reviews have compared CSR with TDC [12, 26–28] or with PDC [29], and two NMAs have evaluated all three techniques [11, 13]. However, these reviews commonly pooled RCTs with non-randomized studies and, in some cases, case reports, an approach that may introduce substantial bias due to differences in study design and methodological rigor [30, 31]. Moreover, none of these reviews formally assessed the certainty of the evidence, limiting the interpretability and reliability of their conclusions.

In terms of findings, prior reviews are generally consistent with our results in suggesting that TDC may be associated with lower blood transfusion requirements compared with CSR [11], and that PDC may not differ significantly from CSR with respect to major adverse events [29]. Additionally, some reviews have reported a shorter hospital stay with TDC compared with CSR [11, 29]. In our analysis, we observed a trend toward a shorter hospital stay with TDC compared with CSR; however, this difference did not reach statistical significance, and the certainty of the evidence was very low, highlighting substantial uncertainty around this association.

Comparisons between TDC and PDC have only been addressed in previous systematic reviews that conducted NMAs [11, 13]. These reviews included only three RCTs each, whereas our review identified seven RCTs. Our findings are broadly consistent with those reported by Li et al. [11].

These discrepancies may be explained by the inclusion of non-randomized studies in previous reviews, which are more susceptible to selection bias, confounding by indication, and preferential allocation of less complex cases to minimally invasive approaches. Such biases may exaggerate apparent benefits in observational analyses that are attenuated when only RCTs are considered.

### Implications

From a clinical perspective, our findings suggest that both TDC and PDC may offer certain perioperative advantages over CSR in selected patients, particularly in terms of reduced blood transfusion requirements and shorter operative time or hospital stay. However, the low to very low certainty of evidence for most outcomes indicates that these potential benefits are very uncertain. Accordingly, clinicians may consider individualizing treatment decisions based on patient age, defect anatomy, institutional expertise, and available resources, rather than relying solely on the presumed advantages of less invasive techniques.

Although some outcomes such as blood loss and operative time showed statistically significant differences, their clinical relevance should be interpreted cautiously [32]. For instance, a reduction of approximately 160 mL in blood loss may be clinically meaningful in smaller children or infants with lower circulating blood volumes [33], while being less impactful in older pediatric patients. Similarly, a reduction of around 90 min in operative time could decrease anesthesia exposure and perioperative risk, but its direct translation into improved recovery or long-term outcomes remains uncertain. Therefore, these findings should be viewed in light of patient characteristics and overall clinical context, rather than statistical significance alone.

### Limitations and strengths

One major limitation is the small number of available RCTs (only seven), all conducted in China, Egypt, or Russia, which may limit the external validity of results to other settings. In addition, 16 potentially eligible studies, predominantly from China, could not be assessed because full texts were unavailable; therefore, we cannot rule out the possibility that some of these reports may have met our inclusion criteria. This limitation may have introduced publication bias; however, we were unable to formally assess it using funnel plots because no meta-analysis included more than 10 studies.

No trials directly compared TDC and PDC groups; thus, this comparison relied solely on indirect estimates with very low certainty. Several comparisons showed high heterogeneity or imprecision, and most clinically relevant outcomes had low or very low certainty of evidence. Moreover, none of the included studies implemented blinding of participants or personnel, which may have influenced the reporting and assessment of outcomes.

Although the transitivity assumption appeared plausible based on the similarity of most clinical characteristics across comparisons, residual intransitivity cannot be completely excluded, particularly due to age differences between study populations. Furthermore, the network structure limited formal assessment of incoherence.

This systematic review with NMA focused exclusively on RCTs, thereby minimizing the risk of bias associated with observational studies. The literature search was comprehensive, with no restrictions on language or publication date, and included major databases such as PubMed, Cochrane Library, and clinical trial registries. Study selection, data extraction, and risk of bias assessment were performed independently and in duplicate by multiple reviewers, enhancing methodological rigor. Additionally, the certainty of the evidence was evaluated using the GRADE approach adapted for NMAs, allowing for a more transparent and structured interpretation of findings.

## Conclusions

Evidence suggests that TDC and PDC may have perioperative advantages over CSR, particularly with respect to blood transfusion and operative-related outcomes. However, no clear differences were observed for major adverse events or residual shunt. The certainty of the evidence was low to very low, indicating that these findings should be interpreted with caution and highlighting the need for well-designed RCTs directly comparing minimally invasive techniques.

## Supplementary Information

Supplementary Material 1.

## Data Availability

All data generated or analysed during this study are included in this published article and its supplementary information files.
