# Supplementary material for: Surgical, perventricular, and transcatheter closure for ventricular septal defects: a network meta-analysis of randomized controlled trials
Source: BMC Cardiovasc Disord. 2026 Mar 28;26:399. doi: 10.1186/s12872-026-05794-w (PMC13151336; doi:10.1186/s12872-026-05794-w)
Supplement: Supplementary file 1 — Supplementary Material 1. [file 12872_2026_5794_MOESM1_ESM.docx]

**Supplementary Material 1. PRISMA 2020 Checklist**

| **Section and Topic** | **Item** | **Checklist item** | **Location in the manuscript** |
| --- | --- | --- | --- |
| **TITLE** | 1 | Identify the report as a systematic review | Title |
| **ABSTRACT** | 2 | See the PRISMA 2020 for Abstracts checklist | Abstract |
| **INTRODUCTION** | 3 | Describe the rationale for the review in the context of existing knowledge | Introduction, paragraphs 1–3 |
|  | 4 | Provide an explicit statement of the objective(s) or question(s) the review addresses | Introduction, last paragraph |
| **METHODS** | 5 | Specify the inclusion and exclusion criteria for the review and how studies were grouped for the syntheses | Methods – Research question and eligibility criteria, paragraphs 1–3 |
|  | 6 | Specify all databases, registers, websites, organisations, reference lists and other sources searched or consulted to identify studies, and the date last searched | Methods – Search strategy, paragraph 1 |
|  | 7 | Present the full search strategies for all databases, registers and websites, including any filters and limits used | Supplementary Material 1 |
|  | 8 | Specify the methods used to decide whether a study met the inclusion criteria, including number of reviewers and whether they worked independently | Methods – Selection and data collection process, paragraph 1 |
|  | 9 | Specify the methods used to collect data from reports, including number of reviewers and whether they worked independently | Methods – Selection and data collection process, paragraph 2 |
|  | 10a | List and define all outcomes for which data were sought | Methods – Variables collected, paragraphs 1–2 |
|  | 10b | List and define all other variables for which data were sought | Methods – Variables collected, paragraph 1 |
|  | 11 | Specify the methods used to assess risk of bias in the included studies | Methods – Risk of bias assessment, paragraphs 1–2 |
|  | 12 | Specify for each outcome the effect measure(s) used in the synthesis | Methods – Statistical analyses, paragraph 1 |
|  | 13a | Describe the processes used to decide which studies were eligible for each synthesis | Methods – Statistical analyses, paragraph 2 |
|  | 13b | Describe methods required to prepare the data for presentation or synthesis | Methods – Statistical analyses, paragraphs 3–4 |
|  | 13c | Describe methods used to tabulate or visually display results | Methods – Statistical analyses, paragraph 2; Results – Tables and figures |
|  | 13d | Describe methods used to synthesize results, models used, heterogeneity assessment, and software | Methods – Statistical analyses, paragraphs 2–4 |
|  | 13e | Describe methods used to explore possible causes of heterogeneity | Methods – Statistical analyses, paragraph 4 |
|  | 13f | Describe any sensitivity analyses conducted | Not performed (not applicable) |
|  | 14 | Describe any methods used to assess risk of bias due to missing results | Methods – Certainty of the evidence assessment, paragraph 2 |
|  | 15 | Describe methods used to assess certainty in the body of evidence | Methods – Certainty of the evidence assessment, paragraphs 1–3 |
| **RESULTS** | 16a | Describe the results of the search and selection process | Results – Study characteristics, paragraph 1; Figure 1 |
|  | 16b | Cite studies that might appear to meet the inclusion criteria but were excluded, and explain why | Results – Study characteristics, paragraph 2; Supplementary Material 2 |
|  | 17 | Cite each included study and present its characteristics | Results – Study characteristics; Table 1 |
|  | 18 | Present assessments of risk of bias for each included study | Results – Risk of bias; Figure 2 |
|  | 19 | Present results of individual studies for all outcomes | Results – Summary of Findings; Table 2; Supplementary material |
|  | 20a | Summarise characteristics and risk of bias among contributing studies | Results – Results and Summary of Findings, paragraph 1 |
|  | 20b | Present results of all statistical syntheses | Results – Summary of Findings; Table 2 |
|  | 20c | Present results of investigations of heterogeneity | Results – Summary of Findings; I² reported where applicable |
|  | 20d | Present results of sensitivity analyses | Not applicable |
|  | 21 | Present assessments of risk of bias due to missing results | Discussion – Limitations and strengths, paragraph 2 |
|  | 22 | Present assessments of certainty of evidence for each outcome | Results – Summary of Findings; Table 2 |
| **DISCUSSION** | 23a | Provide a general interpretation of the results in the context of other evidence | Discussion – Summary of main results |
|  | 23b | Discuss limitations of the evidence included in the review | Discussion – Limitations and strengths |
|  | 23c | Discuss limitations of the review processes used | Discussion – Limitations and strengths |
|  | 23d | Discuss implications for practice, policy, and future research | Discussion – Conclusions |
| **OTHER INFORMATION** | 24a | Provide registration information for the review | Abstract; Methods – Study design and registration |
|  | 24b | Indicate where the review protocol can be accessed | Methods – Study design and registration |
|  | 24c | Describe amendments to protocol | Not applicable (no amendments reported) |
|  | 25 | Describe sources of support and role of funders | Declarations – Funding |
|  | 26 | Declare competing interests | Declarations – Competing interests |
|  | 27 | Report availability of data and materials | Declarations – Availability of data and materials |

**Supplementary Material 2. Search strategy**

Date of last search: February 15, 2026

| Database | Search strategy | Results |
| --- | --- | --- |
| Pubmed / MEDLINE | #1 ("heart septal defects, ventricular"[Mesh] OR "vsd"[TIAB] OR Interventricular[TIAB] OR intraventricular[TIAB] OR Ventricular[TIAB] OR Ventricle[TIAB]) AND (Defect*[TIAB] OR perforation[TIAB] OR (Shunt[TIAB] OR Communication[TIAB]) AND (septal[TIAB] OR septum[TIAB]))  #2 (closure[TIAB] OR close*[TIAB] OR occlu*[TIAB] OR surg*[TIAB] OR repair*[TIAB] OR conventional[TIAB] OR "minimally invasive"[TIAB] OR transcatheter[TIAB] OR percutaneous[TIAB] OR MIPDO[TIAB] OR device[TIAB] OR perventricular[TIAB] OR periventricular[TIAB] OR hybrid[TIAB] OR "mini invasive"[TIAB])  #3 ("Randomized Controlled Trial"[PT] OR "Randomized Controlled Trials as Topic"[Mesh] OR "Clinical Trial"[PT] OR "Clinical Trials as Topic"[Mesh] OR "Clinical Trial, Phase III"[PT] OR "Clinical Trial, Phase II"[PT] OR "Random Allocation"[Mesh] OR "Single-Blind Method"[Mesh] OR random*[TIAB] OR trial[TIAB])  #1 AND #2 AND #3 | 475 |
| Cochrane Library | #1        MeSH descriptor: [Heart Septal Defects, Ventricular] explode all trees  #2        (vsd):ti,ab,kw  #3        (Interventricular):ti,ab,kw  #4        (intraventricular):ti,ab,kw  #5        (ventricular):ti,ab,kw  #6        (ventricle):ti,ab,kw  #7        (defect*):ti,ab,kw  #8        (perforation):ti,ab,kw  #9        (shunt):ti,ab,kw  #10        (communication):ti,ab,kw  #11        (septal):ti,ab,kw  #12        (septum):ti,ab,kw  #13        {OR #1-#6}  #14        {OR #11-#12}  #15        {OR #7-#10}  #16        #14 AND #15  #17        #13 AND #16  #18        (closure):ti,ab,kw  #19        (close*):ti,ab,kw  #20        (occlu*):ti,ab,kw  #21        (surg*):ti,ab,kw  #22        (repair*):ti,ab,kw  #23        (conventional):ti,ab,kw  #24        (minimally invasive):ti,ab,kw  #25        (transcatheter):ti,ab,kw  #26        (percutaneous):ti,ab,kw  #27        (MIPDO):ti,ab,kw  #28        (device):ti,ab,kw  #29        (perventricular):ti,ab,kw  #30        (periventricular):ti,ab,kw  #31        (hybrid):ti,ab,kw  #32        (mini invasive):ti,ab,kw  #33        {OR #18-#32}  #34        #17 AND #33  #35        #17 AND #33 en Ensayos | 522 |
| Embase | #1 ('heart septal defect, ventricular'/exp OR vsd:ti,ab OR interventricular:ti,ab OR intraventricular:ti,ab OR ventricular:ti,ab OR ventricle:ti,ab)  AND (defect*:ti,ab OR perforation:ti,ab OR (shunt:ti,ab OR communication:ti,ab) AND (septal:ti,ab OR septum:ti,ab))  #2 (closure:ti,ab OR close*:ti,ab OR occlu*:ti,ab OR surg*:ti,ab OR repair*:ti,ab OR conventional:ti,ab OR 'minimally invasive':ti,ab OR transcatheter:ti,ab OR percutaneous:ti,ab OR MIPDO:ti,ab OR device:ti,ab OR perventricular:ti,ab OR periventricular:ti,ab OR hybrid:ti,ab OR 'mini invasive':ti,ab)  #3 ('randomized controlled trial'/exp OR 'randomized controlled trial (topic)'/exp OR 'clinical trial'/exp OR 'clinical trial (topic)'/exp OR 'clinical trial phase 3'/exp OR 'clinical trial phase 2'/exp OR 'random allocation'/exp OR 'single blind method'/exp OR random*:ti,ab OR trial:ti,ab)  #1 AND #2 AND #3 | 1119 |
| Clinicaltrials.gov | Ventricular Septal Defects OR VSD  Filter: Child (birth - 17) | 67 |
| Google Scholar | ventricular septal defect closure randomized trial | 100 first results |

**Supplementary Material 3. Studies excluded after full text review.**

| **Author (year)** | **Title** | **Exclusion reasons** |
| --- | --- | --- |
| Bacha (2005) | Multicenter experience with perventricular device closure of muscular ventricular septal defects | No comparison group |
| Arora (2003) | Transcatheter closure of congenital ventricular septal defects: experience with various devices | No comparison group |
| Kozlik-Feldmann (2021) | Long-term outcome of perimembranous VSD closure using the Nit-Occlud® Lê VSD coil system | No randomization |
| Fu (2016) | Transcatheter closure of perimembranous ventricular septal defects using the new Amplatzer Membranous VSD Occluder: Results of the U.S. phase I trial | No randomization |
| Esteves (2012) | Occlusion of the perimembranous ventricular septal defect using CERA® devices | No randomization |
| NR | Transcatheter closure of perimembranous VSD with the Amplatzer ductal occluder - First trial in Korea | No randomization |
| Cheng (2013) | Totally endoscopic congenital heart surgery compared with the traditional heart operation in children | No intervention of interest |
| Gong (2024) | Midterm follow-up results of implantation of a fully biodegradable ventricular septal defect occluder | Cohort study |
| Koneti (2024) | Transcatheter Closure of Perimembranous Ventricular Septal Defect Using KONAR-MF™: A Multicenter Experience | Cohort study |
| Kuswiyanto (2022) | Transcatheter Closure of Perimembranous Ventricular Septal Defect Using the Lifetech Konar-Multi Functional Occluder: Early to Midterm Results of the Indonesian Multicenter Study | Cohort study |
| Zhang (2020) | A comparative study on the efficacy of transthoracic minimal invasive closure and surgery in pediatric patients with ventricular septal defect | Abstract paper |
| Haas (2017) | Interventional VSD-Closure with the Nit-Occlud® Lê VSD-Coil in 110 Patients: Early and Midterm Results of the EUREVECO-Registry | Cohort study |
| Hua (2016) | Transcatheter closure of perimembranous ventricular septal defects with the Amplatzer Vascular Plug-II | Case report |
| Chen (2015) | Transcatheter Closure of Intracristal Ventricular Septal Defect With Mild Aortic Cusp Prolapse Using Zero Eccentricity Ventricular Septal Defect Occluder | Cohort study |
| Hijazi (2000) | Transcatheter closure of single muscular ventricular septal defects using the amplatzer muscular VSD occluder: initial results and technical considerations | Case series |
| NR | Perimembranous VSD closure using the new CERA® devices | Poster publication |
| Gu (2010) | Transcatheter closure of perimembranous ventricular septal defects using a novel wire-maintaining technique | No population of interest |
| Li (2017) | Comparative analysis of the success rate, complications and serum inflammatory factors in patients with congenital heart disease treated by interventional therapy and traditional surgical treatment | No separate information |
| Luo (2001) | Ministernotomy versus full sternotomy in congenital heart defects: a prospective randomized study | No separate information |
| Yang (2013) | Randomized, controlled trial of transcatheter versus surgical closure of perimembranous ventricular septal defects in children | Same study as Yang (2014) |

**Supplementary Material 4. Pair-wise meta-analyses. For informational purposes only, as the analyses reported in the manuscript are those from the network meta-analysis.**

| *Outcomes* | *Meta-analyses* |
| --- | --- |
| *Complete closure* | ***Command: “random OR”***  ***(dersimonian laird):***  *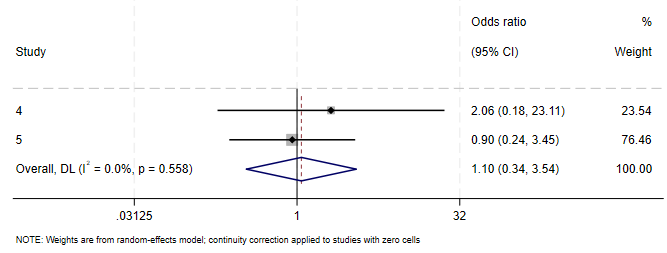*  *random or: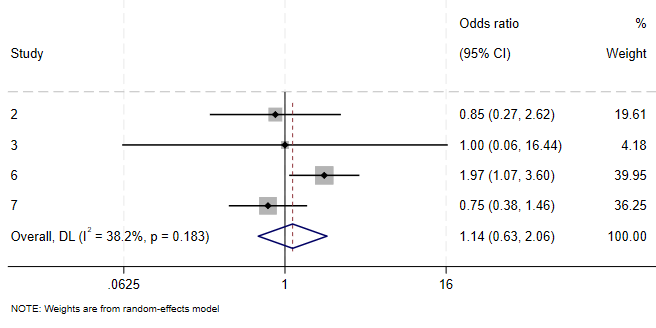*  *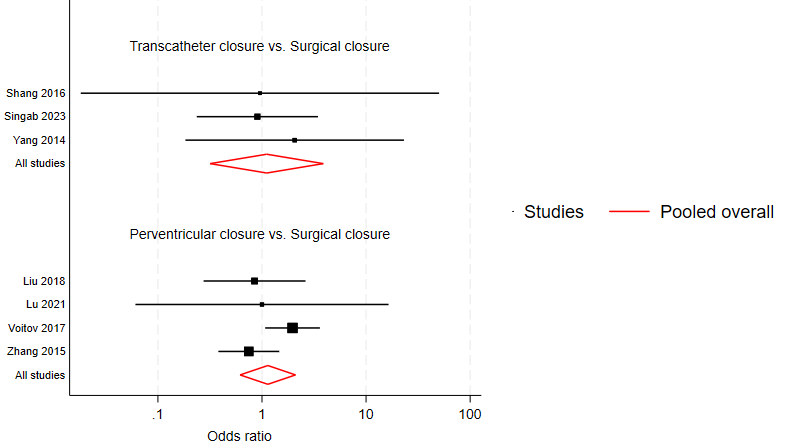*  *Network meta-analysis**  *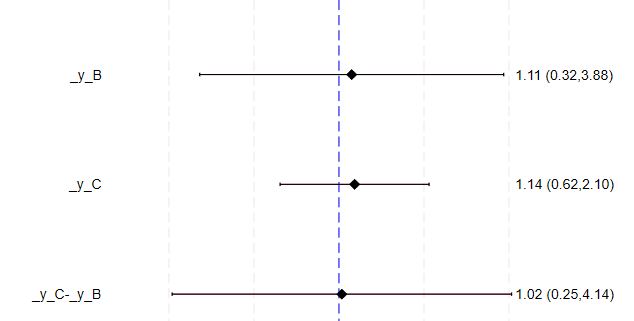* |
| *Major adverse events* | *Transcatheter closure vs Surgical closure*  *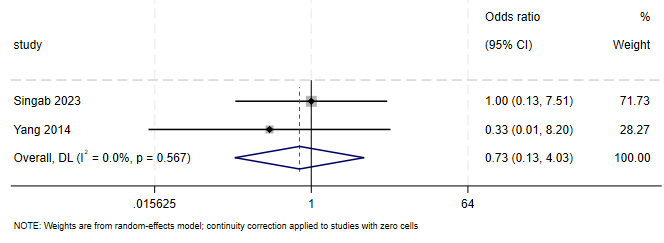*  *Perventricular closure vs Surgical closure*  *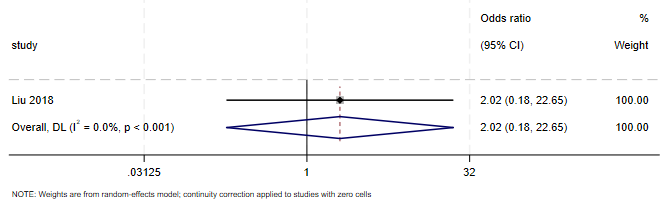*  *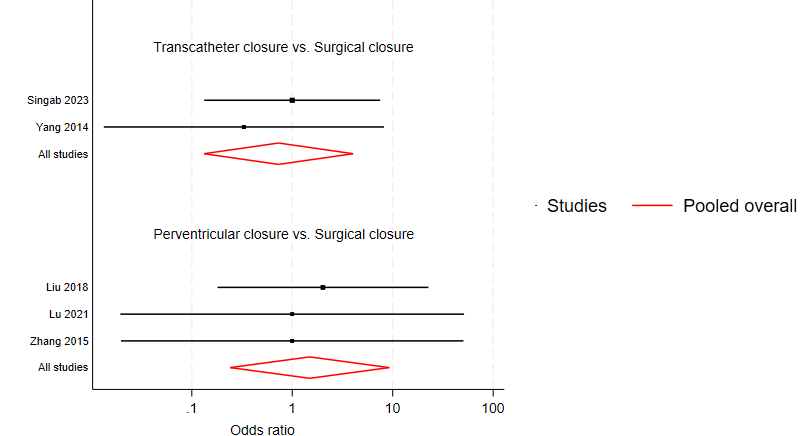*  *Network meta-analysis**  *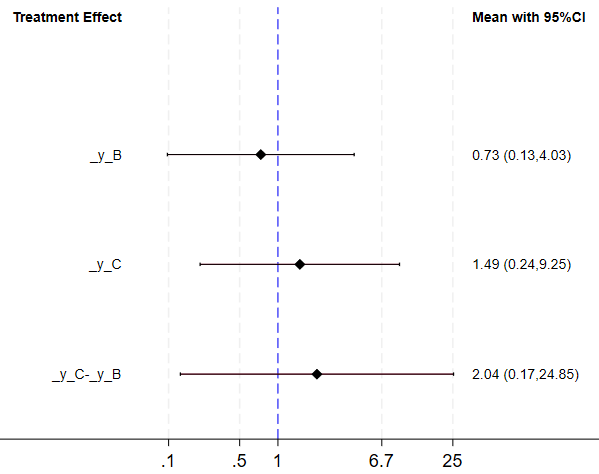* |
| *Minor adverse events* | *Transcatheter closure vs Surgical closure*  *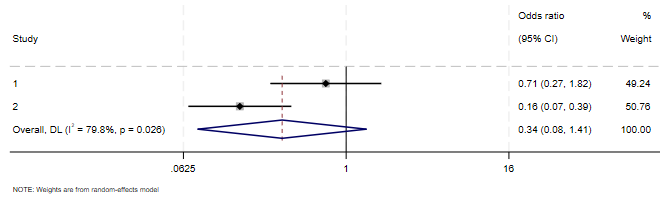* |
| *Tricuspid regurgitation* | *Transcatheter closure vs Surgical closure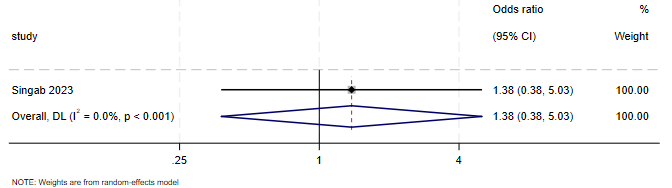*  *Perventricular closure vs Surgical closure*  *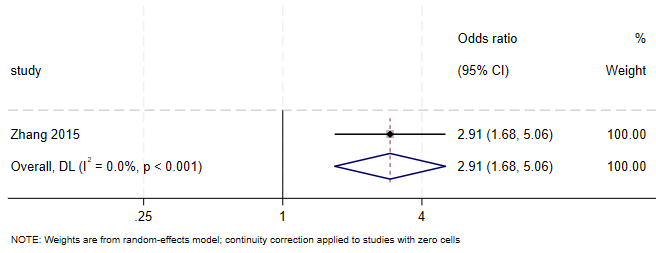*  *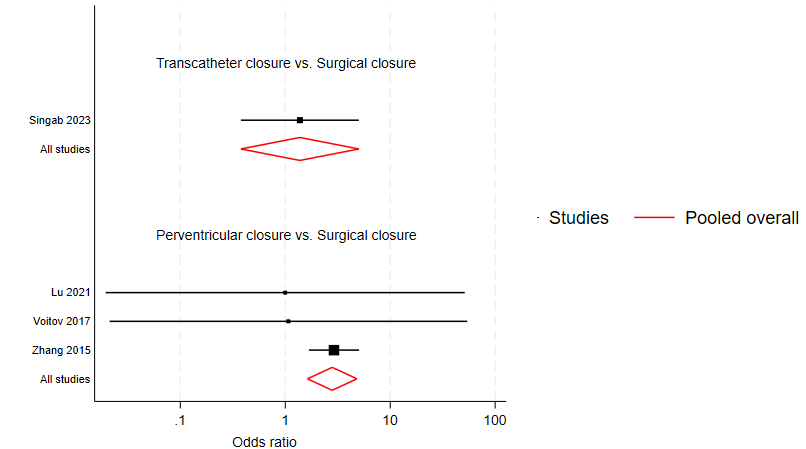*  *Network meta-analysis**  *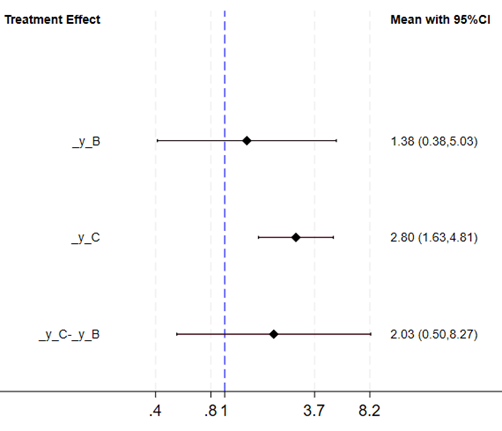* |
| *Blood transfusion* | *Transcatheter closure vs Surgical closure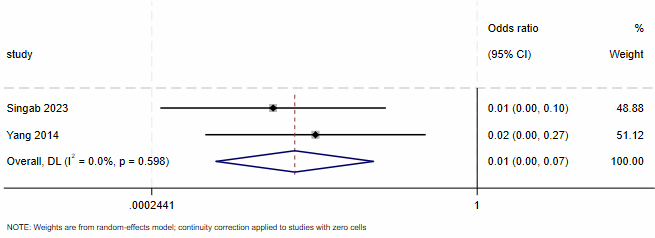*  *Perventricular closure vs Surgical closure*  *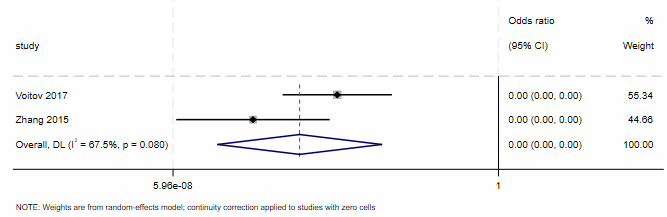*  *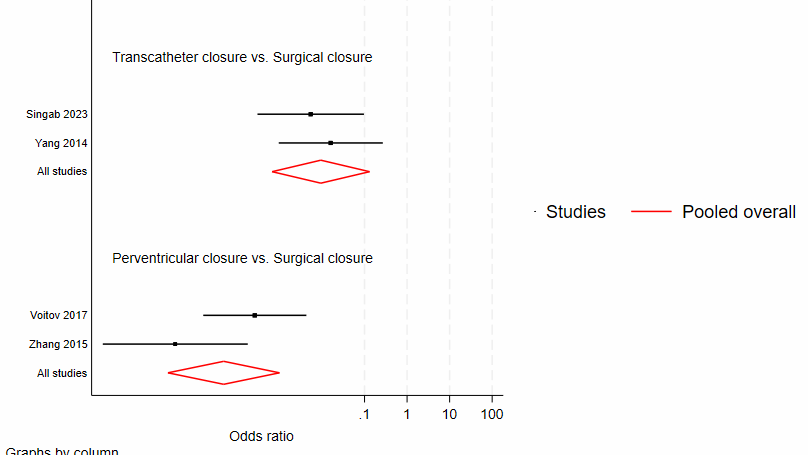*  *Network meta-analysis**  *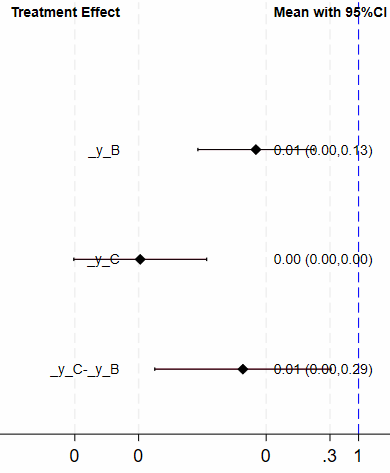* |
| *Blood loss* | *Transcatheter closure vs Surgical closure*  *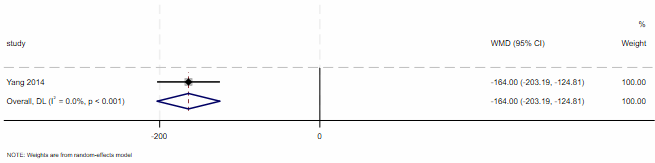*  *Perventricular closure vs Surgical closure*  *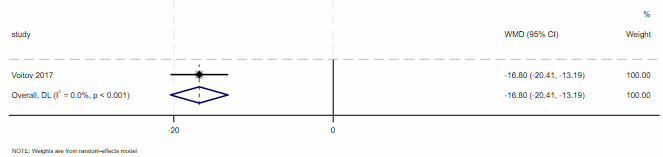*  *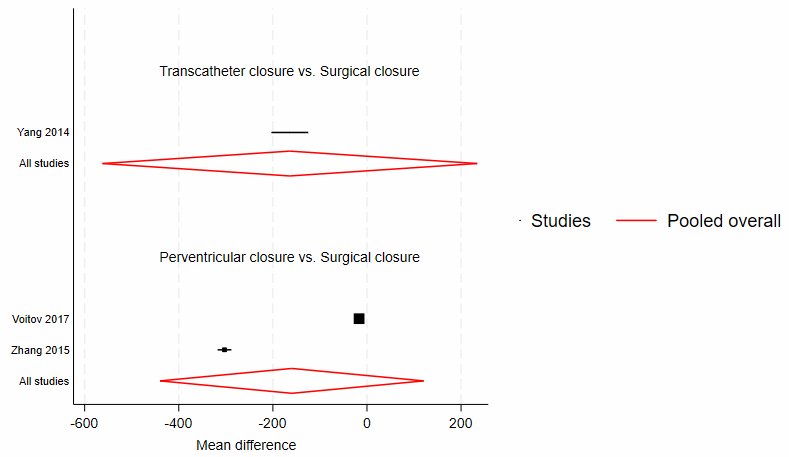*  *Network meta-analysis**  *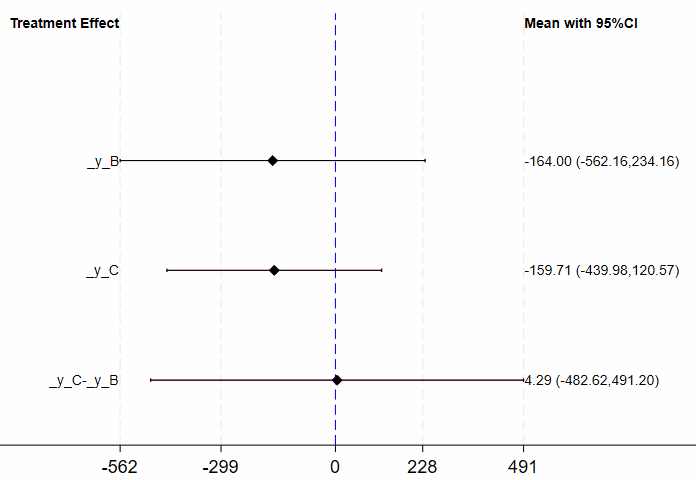* |
| *Hospital stay* | *Transcatheter closure vs Surgical closure*  *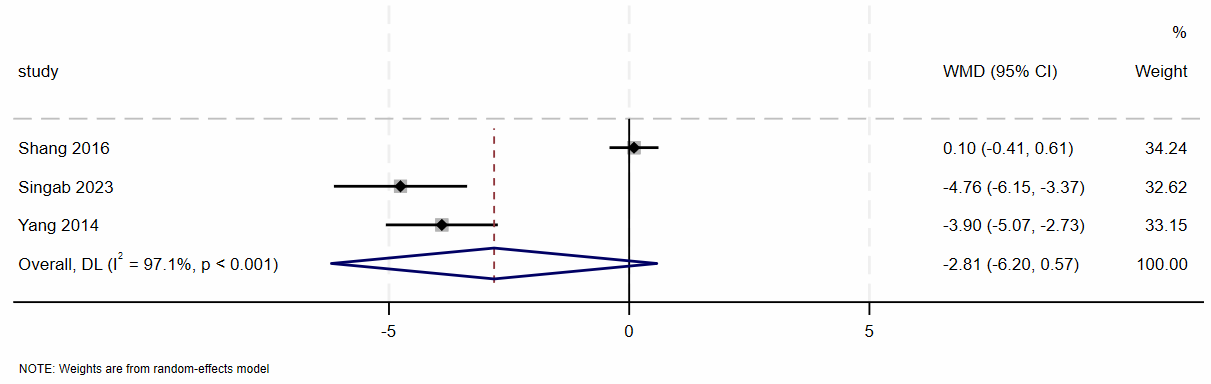*  *Perventricular closure vs Surgical closure*  *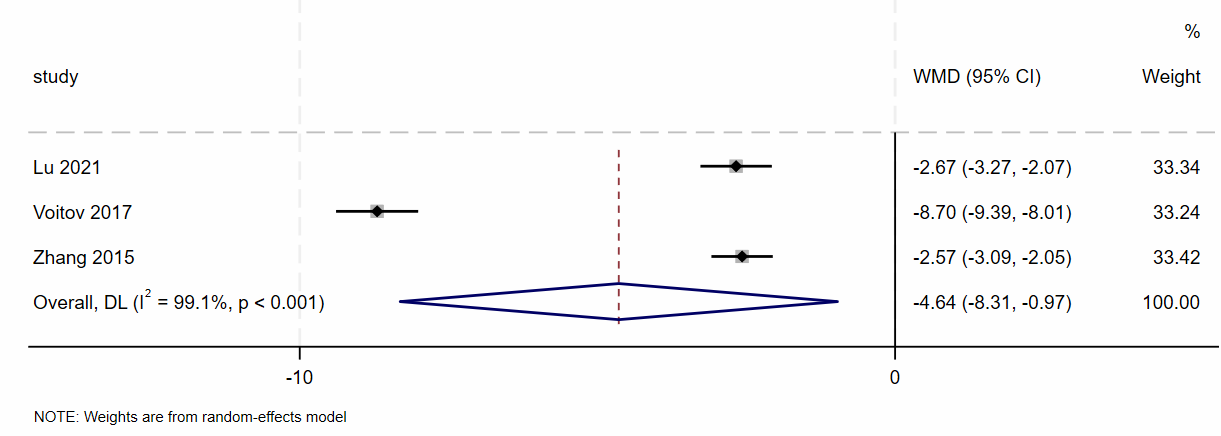*  *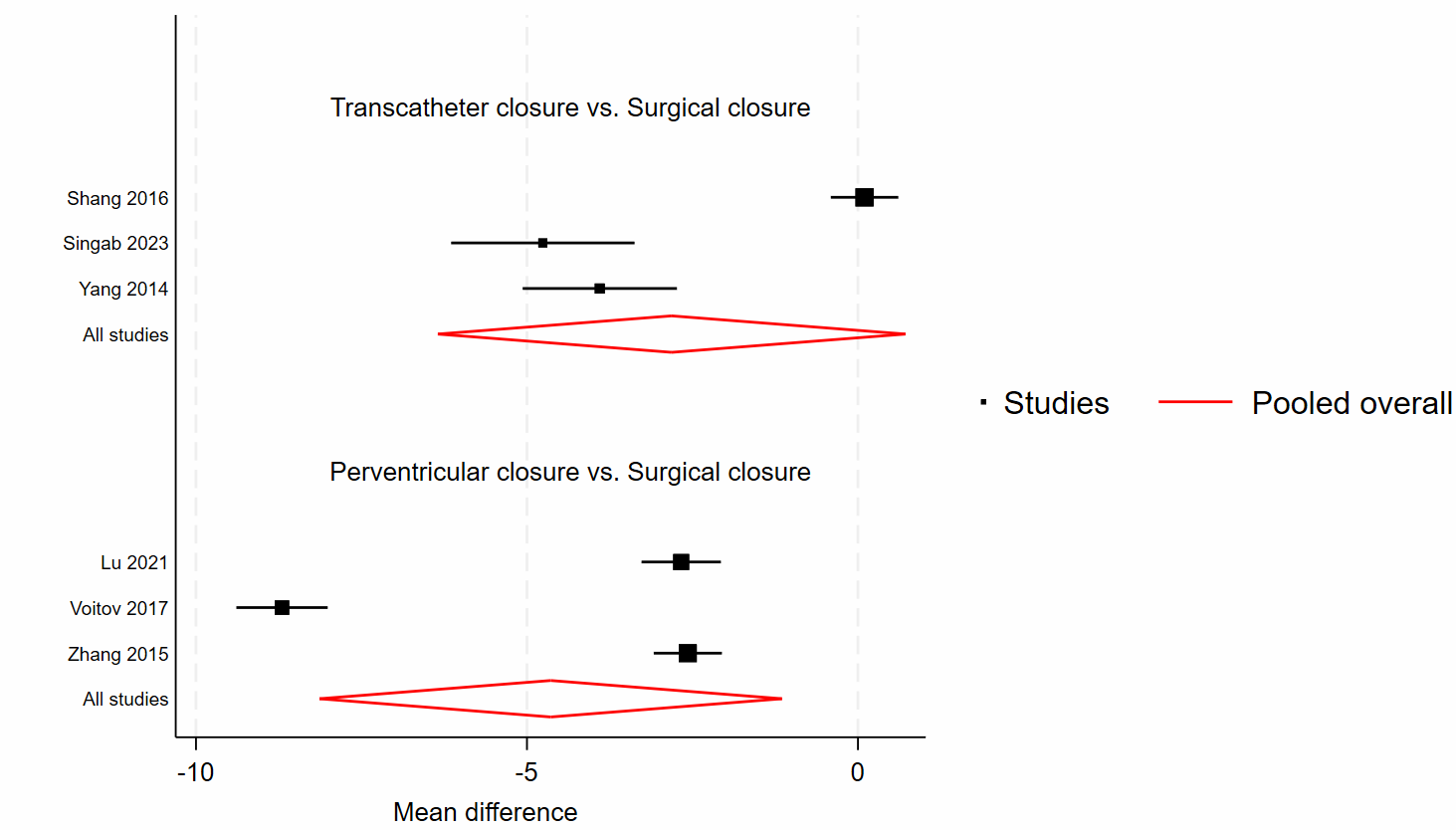*  *Network meta-analysis**  *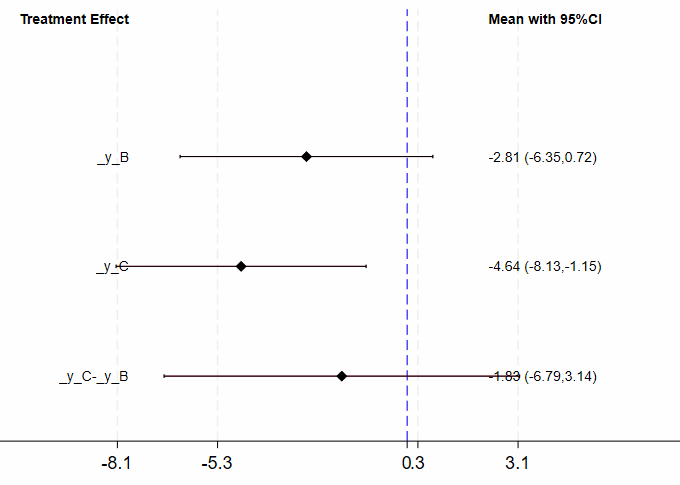* |
| *Operation time* | *Transcatheter closure vs Surgical closure*  *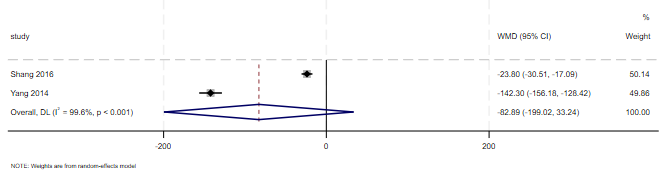*  *Perventricular closure vs Surgical closure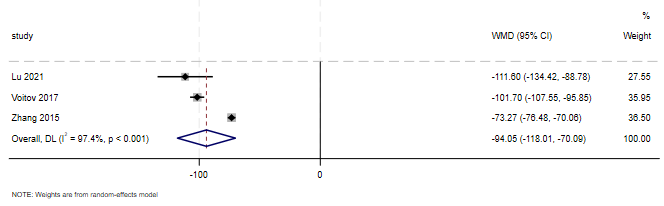*  *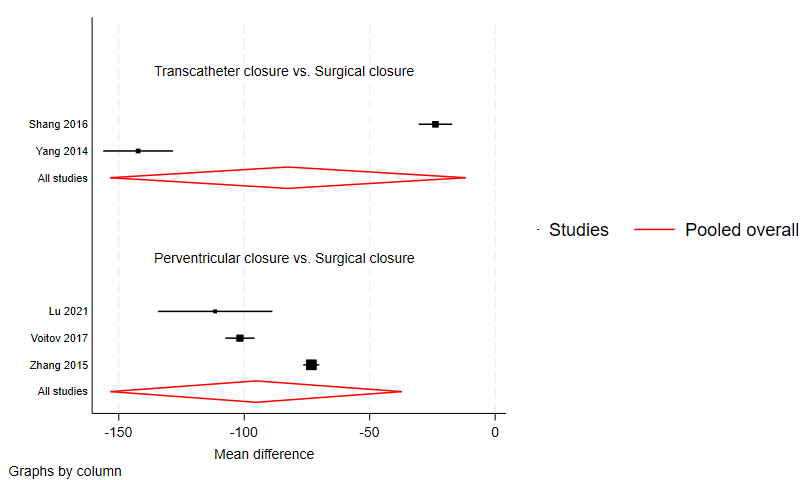*  *Network meta-analysis**  *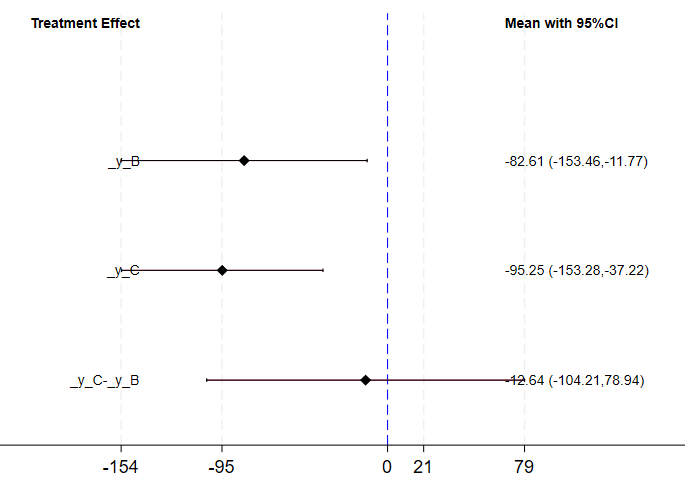* |

**_y_B: trans vs surg*

*_y_C: perv vs surg*

*_y_C-_y_B: perv vs trans*

**Supplementary Material 5. Network plots for all outcomes**

| Complete closure:  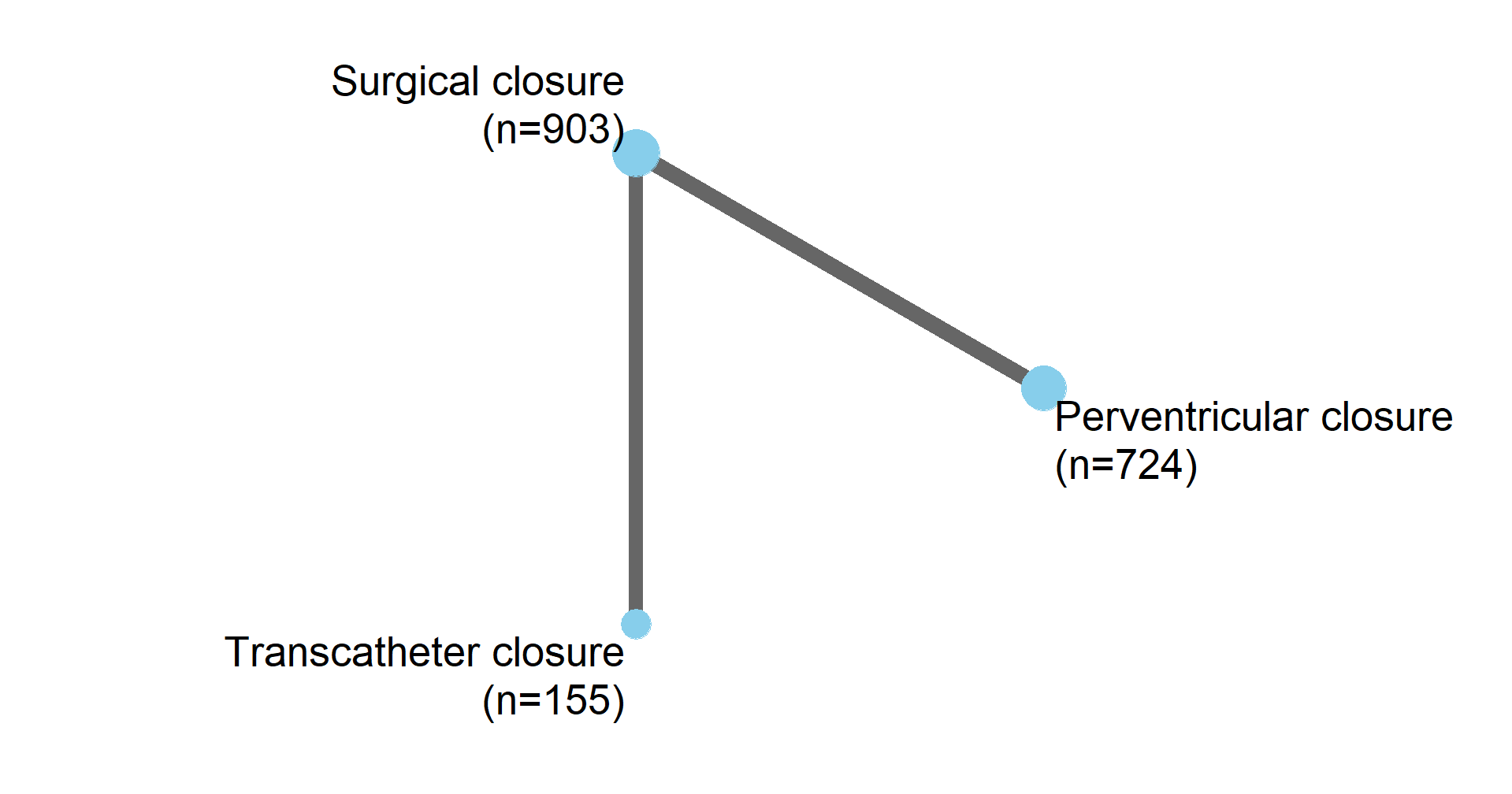 | Minor adverse events:  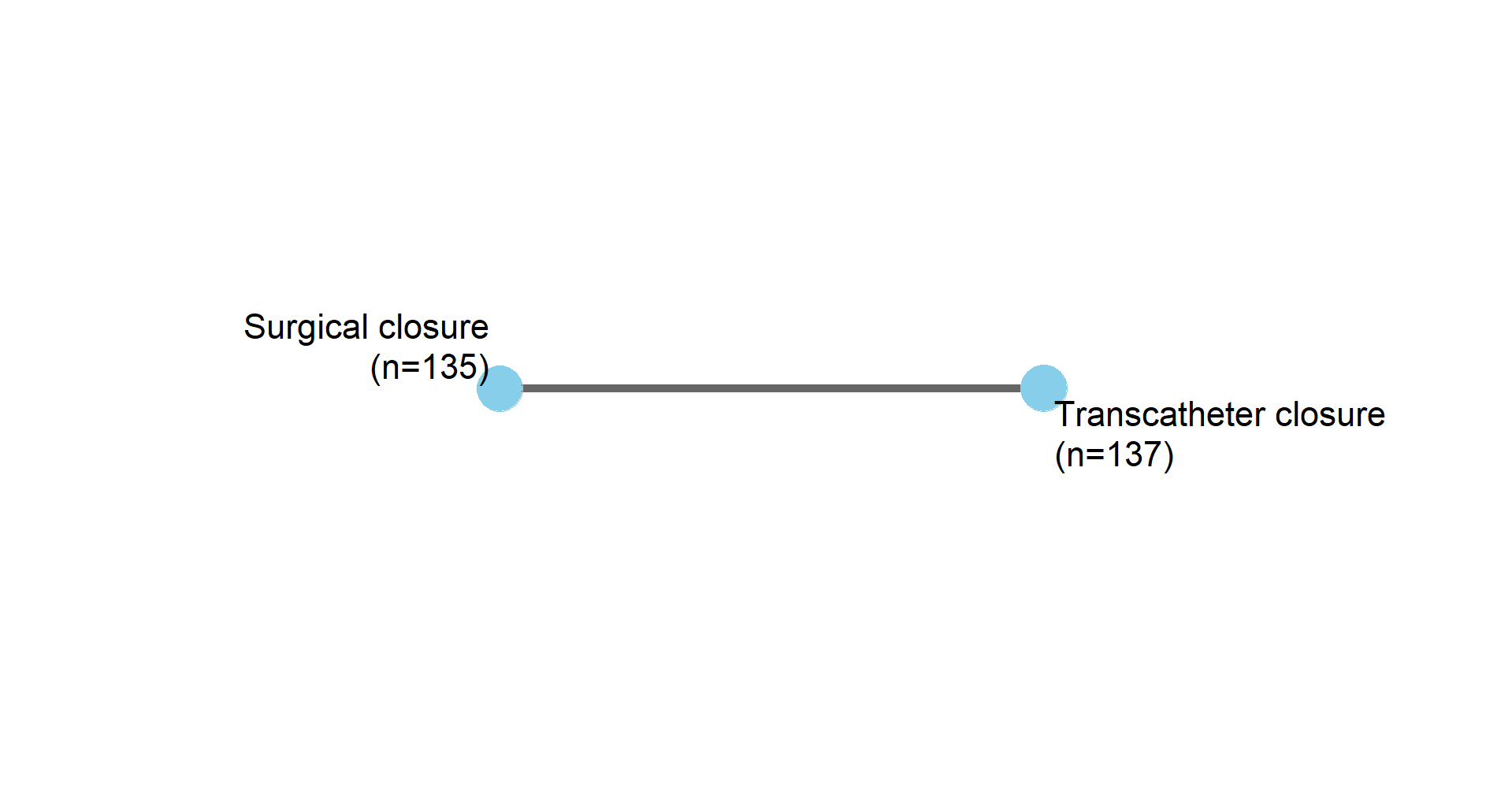 |
| --- | --- |
| Blood loss  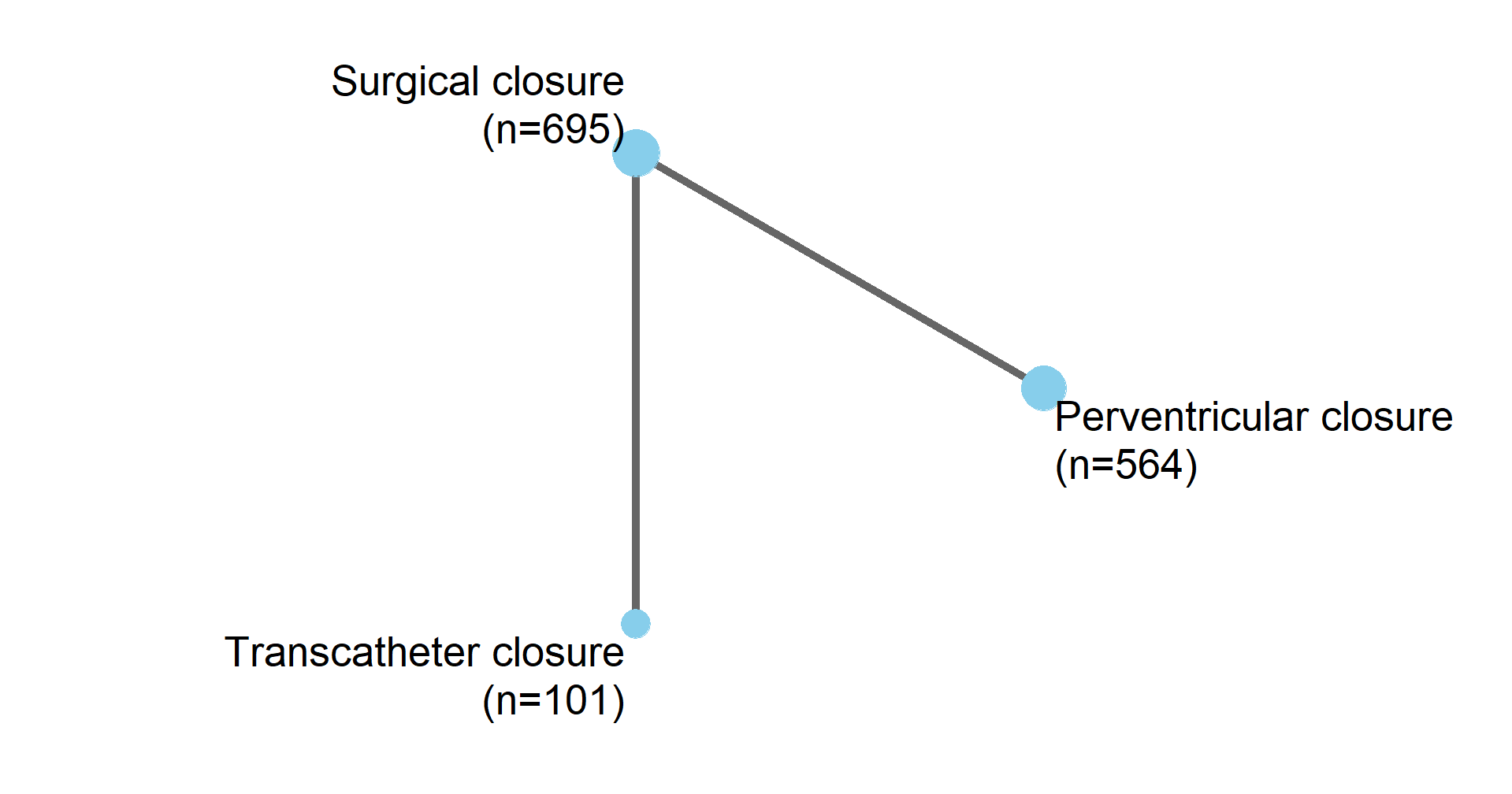 | Hospital stay:  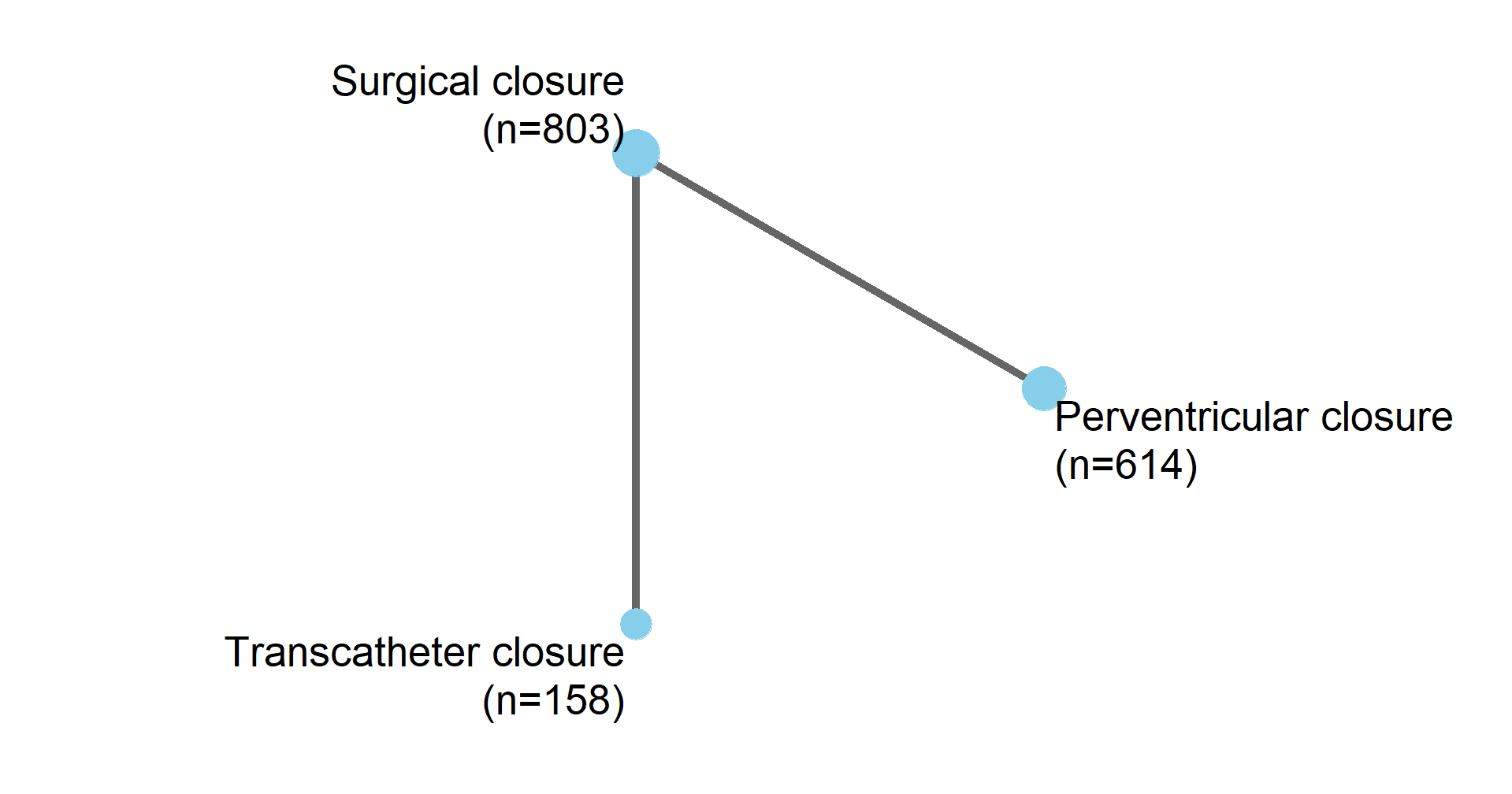 |
| Operative time:  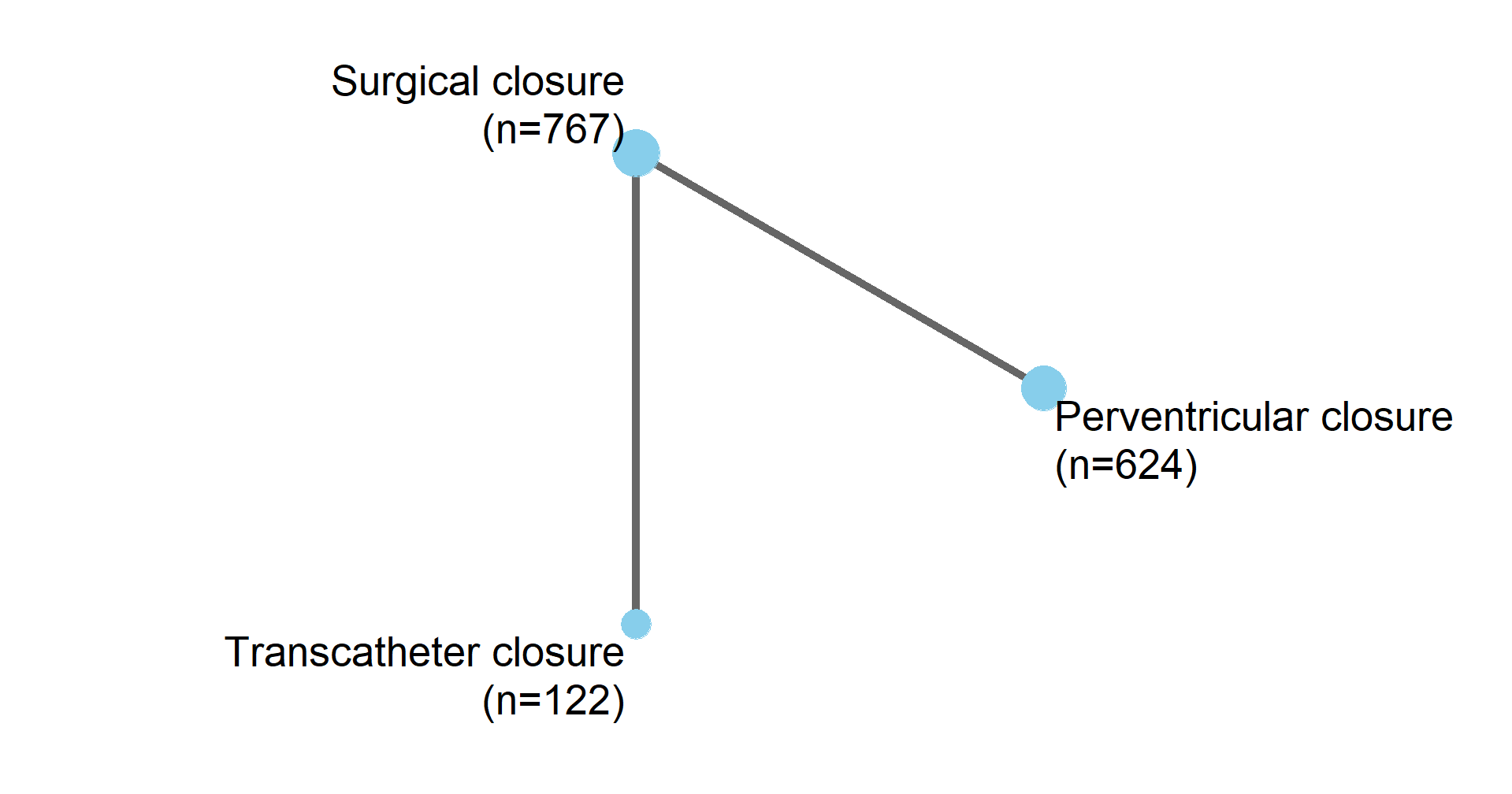 | Transfusion:  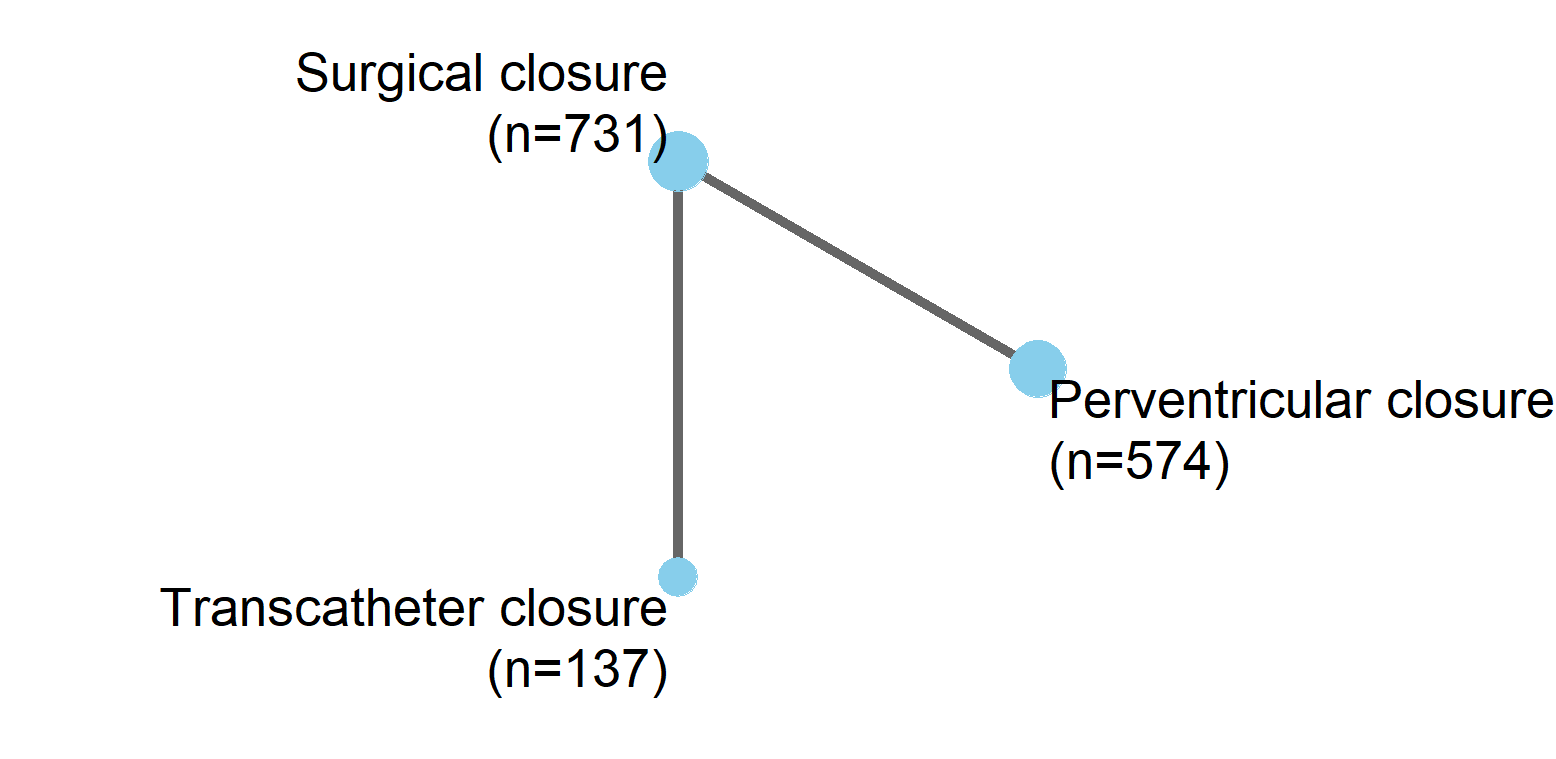 |
| Cardiac failure:  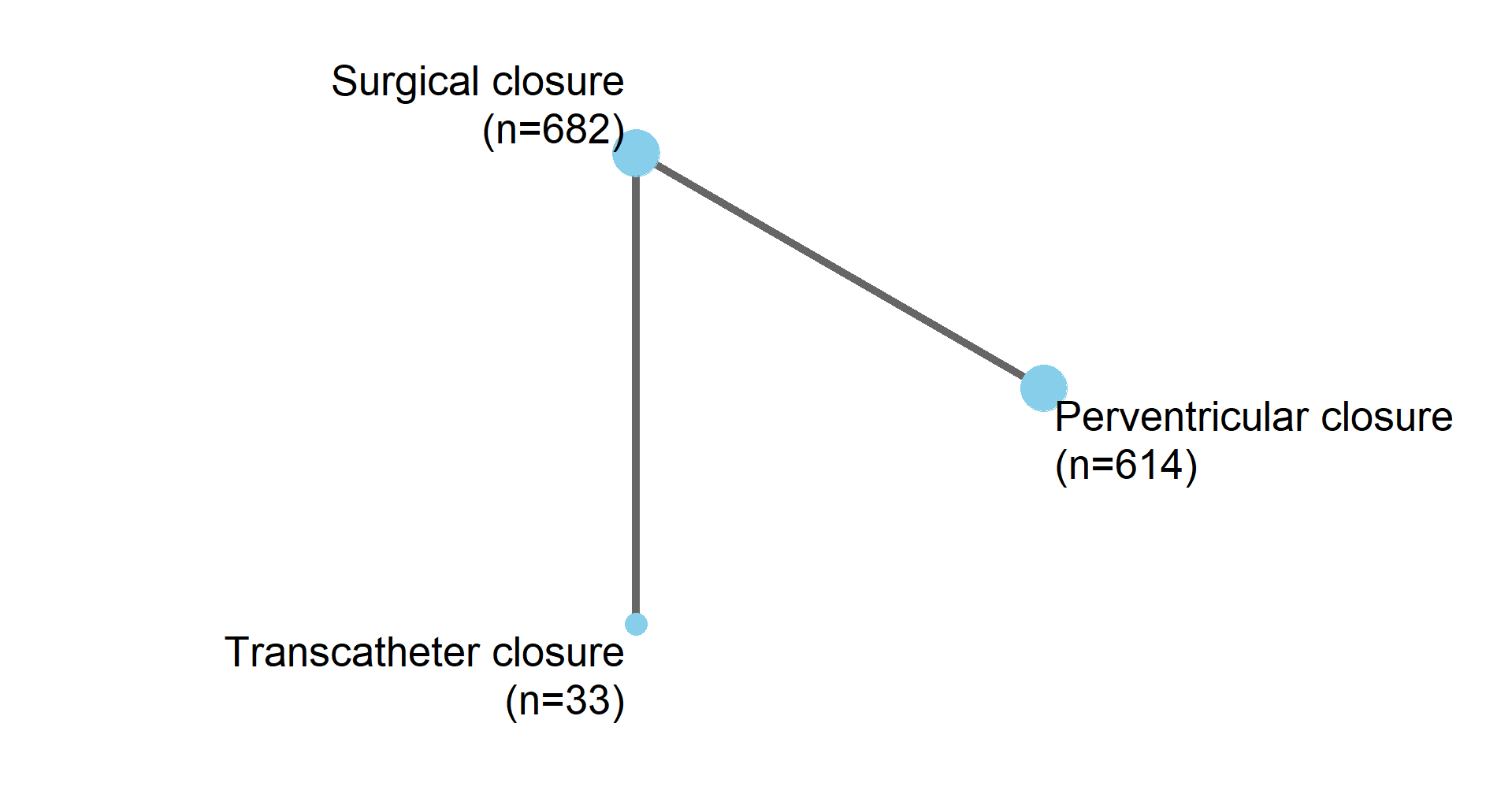 | Major events:  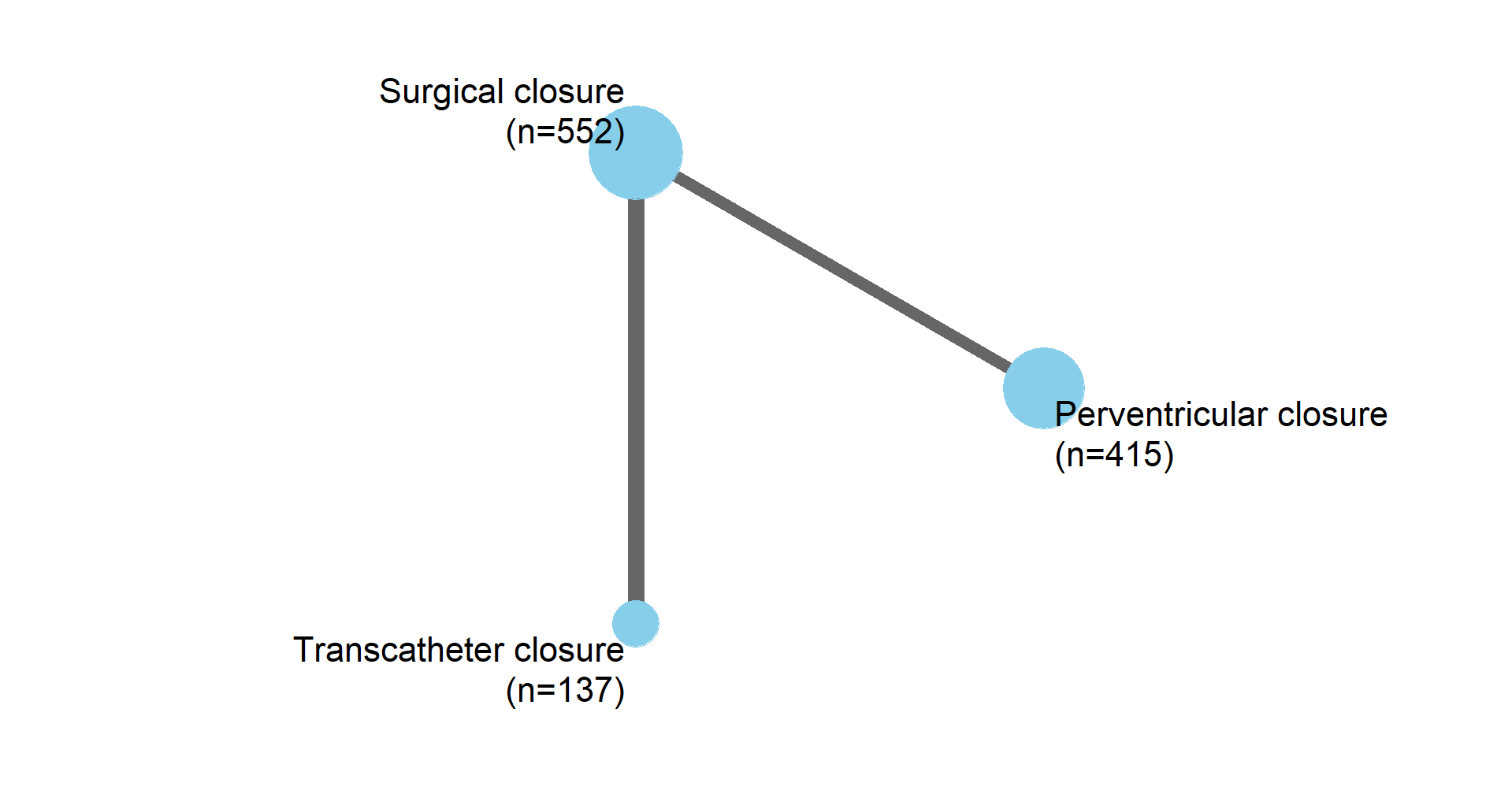 |

**Supplementary Material 6. Criteria to classify major and minor adverse events across studies**

|  | **Major adverse events** | **Minor adverse events** |
| --- | --- | --- |
| Liu 2018 | Composite of all-cause death in the intensive care unit and hospitalisation, cardiac-specific death, sudden cardiac arrest, stroke, complete atrioventricular block requiring pacemaker implantation or reoperation, more than moderate regurgitation requiring surgery, acute kidney injury requiring dialysis, Adams–Stokes syndrome, low cardiac output syndrome, pulmonary hypertensive crisis, reoperation for major bleeding or wound infection and acute respiratory distress syndrome. | Composite of insignificant arrhythmia, transient ischaemic attack, acute kidney injury not requiring dialysis, trace to mild valve regurgitations, procedure- specific events as well as device-only complications |
| Lu 2021 | All-cause death, cardiac-specific death, cardiac perforation, cardiac tamponade, iatrogenic tricuspid regurgitation, iatrogenic aortic regurgitation, dislocation or distortion of occluders and severe hemolysis |  |
| Yang 2014 | Death, complete atrioventricular block, new onset valvular regurgitation that required surgical repair, thoracic re exploration | Vascular complications at the puncture site, new or increased valvular regurgitation less than two grades, blood loss requiring transfusion, and first- or second-degree heart block without any long-term complications. |
| Singab 2023 | Late death due to a cause in direct relation to the procedure, thromboembolic events, repeating the operation, new onset severe valvular regurgitation that requires surgical intervention, device detachment, and the need for a permanent pacemaker. | Wound complications that required hospital admission or surgical debridement, reopening due to bleeding in the surgical group, groin hematoma in the transcatheter group, new arrhythmia, peripheral ischemia that not required endovascular intervention, increased one or two degrees of valvular regurge that not required intervention, self-limited hemolysis due to small residual, any efusion that required drainage as with chest tube or aspiration. |
| Zhang 2016 | Serious life-threatening complications |  |
